# The type VI secretion system of the emerging pathogen *Stenotrophomonas maltophilia* complex has antibacterial properties

**DOI:** 10.1128/msphere.00584-23

**Published:** 2023-11-17

**Authors:** Cristian V. Crisan, Daria Van Tyne, Joanna B. Goldberg

**Affiliations:** 1 Division of Pulmonary, Asthma, Cystic Fibrosis, and Sleep, Department of Pediatrics, Emory University School of Medicine, Atlanta, Georgia, USA; 2 Emory+Children’s Center for Cystic Fibrosis and Airway Disease Research, Emory University School of Medicine, Atlanta, Georgia, USA; 3 Division of Infectious Diseases, University of Pittsburgh School of Medicine, Pittsburgh, Pennsylvania, USA; University of Kentucky College of Medicine, Lexington, Kentucky, USA

**Keywords:** *Stenotrophomonas maltophilia*, type VI secretion system, competition, antibacterial

## Abstract

Antagonistic behaviors between bacterial cells can have profound effects on microbial populations and disease outcomes. Polymicrobial interactions may be mediated by contact-dependent proteins with antibacterial properties. The type VI secretion system (T6SS) is a macromolecular weapon used by Gram-negative bacteria to translocate proteins into adjacent cells. Many pathogens deploy the T6SS to escape immune cells, eliminate commensal bacteria, and facilitate infection. *Stenotrophomonas maltophilia* complex is a Gram-negative opportunistic pathogen that primarily affects immunocompromised people and infects the lungs of patients with cystic fibrosis. Infections with the bacterium can be deadly and are challenging to treat because many isolates are multidrug-resistant. We found that globally dispersed *S. maltophilia* complex clinical and environmental strains possess T6SS genes. We demonstrate that the T6SS of an *S. maltophilia* complex patient isolate is active and can eliminate other bacteria. We provide evidence that the T6SS contributes to the competitive fitness of *S. maltophilia* complex against a co-infecting *Pseudomonas aeruginosa* isolate, and the T6SS alters the organization of *S. maltophilia* and *P. aeruginosa* co-cultures. This study expands our knowledge of the mechanisms employed by *S. maltophilia* complex to secrete antibacterial proteins and compete against other bacteria.

**IMPORTANCE:**

Infections with the opportunistic pathogen *Stenotrophomonas maltophilia* complex can be fatal for immunocompromised patients. The mechanisms used by the bacterium to compete against other prokaryotes are not well understood. We found that the type VI secretion system (T6SS) allows *S. maltophilia* complex to eliminate other bacteria and contributes to the competitive fitness against a co-infecting isolate. The presence of T6SS genes in isolates across the globe highlights the importance of this apparatus as a weapon in the antibacterial arsenal of *S. maltophilia* complex. The T6SS may confer survival advantages to *S. maltophilia* complex isolates in polymicrobial communities in both environmental settings and during infections.

## INTRODUCTION

*Stenotrophomonas maltophilia* is an emerging, globally dispersed opportunistic pathogen that causes infections of the lungs, brain, gastrointestinal tract, nervous system, eyes, blood, skin, and bones (1–3). Bacteremia caused by this bacterium can have mortality rates as high as 65% (4). Infections are difficult to treat because many isolates are intrinsically resistant to multiple antibiotics, including cephalosporins (5–7). In some hospitals, *S. maltophilia* is the most common Gram-negative, carbapenem-resistant bacterium in patients with bacteremia (8). In 2018, the World Health Organization listed *S. maltophilia* as an important pathogen for which novel antibiotic treatments are urgently needed (6, 7).

Immunocompromised people and cancer patients are especially susceptible to *S. maltophilia* (4, 9). Cystic fibrosis (CF), a genetic disease that affects more than 150,000 people worldwide, leads to chronic pulmonary bacterial infections (10). Approximately 10%–30% of CF patients harbor *S. maltophilia* in their lungs at least once during their life (11, 12). CF pulmonary *S. maltophilia* infections can be associated with up to threefold higher mortality, more severe exacerbations, and an increased risk of requiring lung transplants (13). The bacterium is also detected in sputum from COVID-19 patients and has the highest rates of multidrug resistance among bacterial species from this population (14–16).

*S. maltophilia* is ubiquitously found in water and soil environments and has been isolated from hospital surfaces and medical devices (17, 18). The species exhibits extensive genomic diversity and has been subdivided into 23 monophyletic lineages (19). Due to its diversity, the phylogenetic classification of the species is problematic, and Gröschel et al. proposed the term “*S. maltophilia* complex” for isolates that are identified as *S. maltophilia* by diagnostic procedures (19).

*S. maltophilia* complex strains are often isolated from patients with other co-infecting pathogens like *Pseudomonas aeruginosa*, and both cooperative and antagonistic interactions have been observed between these two bacterial species (20–24). The *S. maltophilia* K279a blood isolate possesses a type IV secretion system (T4SS) with antibacterial properties (20, 21, 24). The T4SS contributes to the ability of K279a to eliminate other bacteria, including clinical *P. aeruginosa* isolates (20, 21, 24). VirB10 (part of the outer membrane pore complex) and VirD4 (an ATPase) proteins are required for the antibacterial activity of the T4SS in *S. maltophilia* K279a (20, 21, 24).

The type VI secretion system (T6SS) is an important proteinaceous macromolecular apparatus used by many Gram-negative pathogens to deliver toxic proteins (effectors) into target cells (25–27). A membrane complex (which includes the essential TssM protein) and a baseplate structure assemble within the membrane of cells that possess an active T6SS (28, 29). The inner tube of stacked Hcp (hemolysin-coregulated protein) hexamers is surrounded by a contractile outer sheath formed by TssB and TssC proteins (30–32). Previous studies used TssC protein sequences to determine T6SS phylogenetic relationships (33). T6SS loci can be divided into four types (i, ii, iii, and iv), while type i can be further divided into six subtypes (i1, i2, i3, i4a, i4b, and i5) (34–37). Contractions of the outer sheath facilitate excretion of the Hcp tube, which is capped by a VgrG (valine-glycine repeat protein) trimer and a PAAR (proline-alanine-alanine-arginine) protein complex (38–40). Secreted T6SS toxic proteins (effectors) interact with VgrG, PAAR, or Hcp proteins and can exhibit both antibacterial and anti-eukaryotic properties (25, 35, 40–42). Rhs (rearrangement hotspot) toxins are large, polymorphic proteins that may associate with the T6SS (43, 44). Antibacterial effectors exert their toxicity on the cell envelope (where they can damage the peptidoglycan layer, degrade lipid membranes, or form pores) and the cytoplasm (where they can alter nucleotides or interfere with protein synthesis) of competitor bacteria (45–51).

In this study, we performed bioinformatic searches using NCBI databases and identified *S. maltophilia* complex isolates from both patient and environmental sources that possess T6SS-encoding genes. We found that a clinical *S. maltophilia* complex strain encodes a T6SS that is active under standard laboratory conditions and can eliminate other bacteria like *Escherichia coli* and *Burkholderia cenocepacia*. We also observed that the T6SS confers a competitive advantage to this *S. maltophilia* against a co-infecting *P. aeruginosa* strain obtained from the same patient. We used confocal microscopy to determine that the T6SS alters the spatial organization of co-cultures containing both *S. maltophilia* and *P. aeruginosa*. The presence of T6SS-encoding genes in *S. maltophilia* complex isolates across the globe highlights the importance of this apparatus as a weapon in the antibacterial arsenal of *S. maltophilia*. The T6SS may confer survival advantages to *S. maltophilia* complex strains in polymicrobial communities in environmental settings and during infections.

## RESULTS

### T6SS genes are found globally distributed in *S. maltophilia* complex patient and environmental isolates

**Fig 1:**
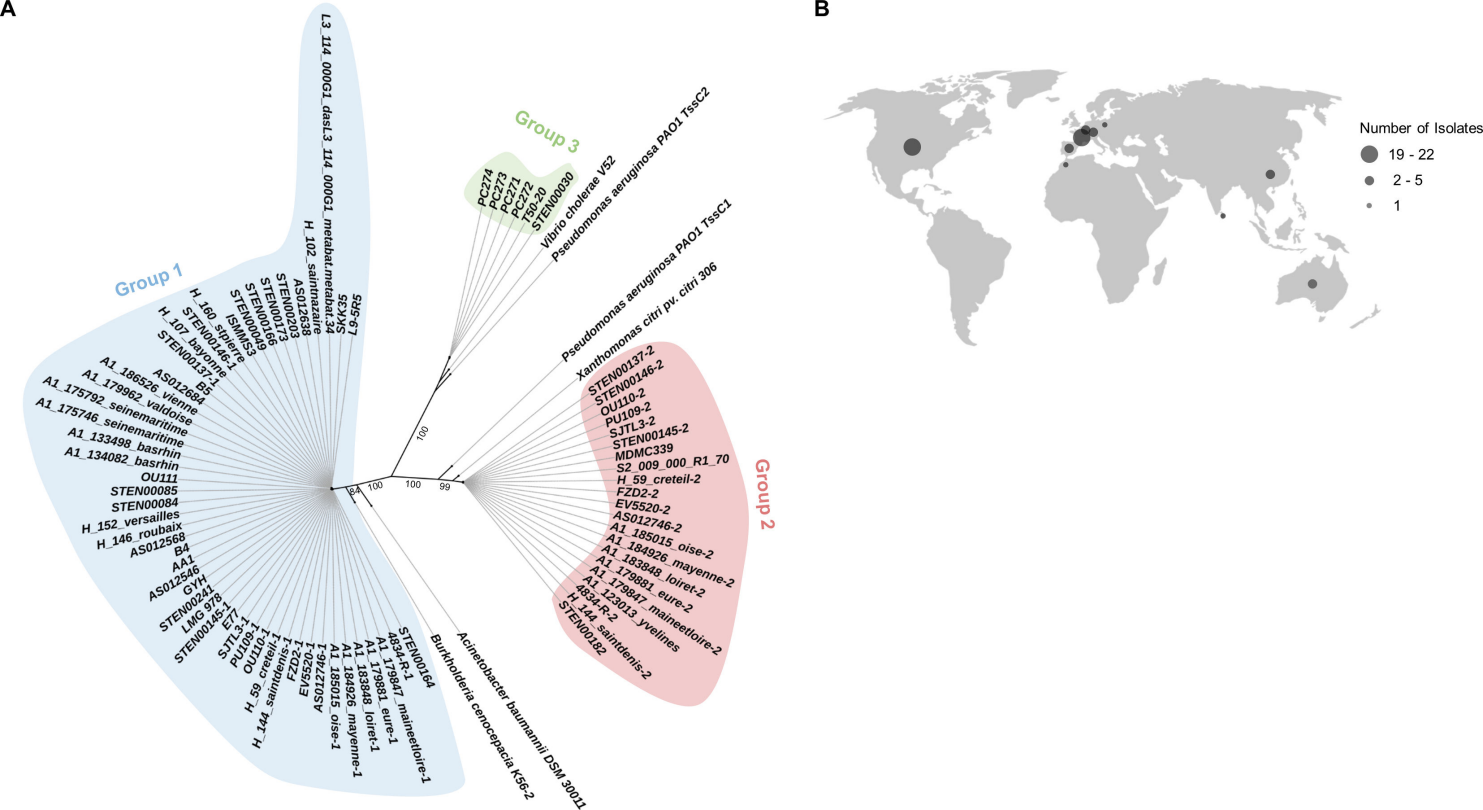
Patient and environmental *S. maltophilia* complex isolates from diverse geographic locations harbor T6SS-encoding genes. (**A**) A BlastP search was conducted to identify *S. maltophilia* complex TssC proteins. TssC sequences of the indicated strains were aligned, and a phylogenetic tree was constructed. Branch titles designate the strain name that encodes the respective TssC homolog. Branch numbers indicate support values. Strains that harbor a TssC from groups 1 and 2 are indicated by a “−1” or “−2,” respectively, after the strain name. (**B**) The number of *S. maltophilia* complex isolates with T6SS-encoding genes from each country is displayed.

To analyze the distribution of T6SS-encoding genes in *S. maltophilia* isolates, we used the sequence of the TssC sheath protein from *Xanthomonas citri* to search among approximately 1,000 *S*. *maltophilia* complex genomes from RefSeq and GenBank databases for homologous proteins (52, 53). *X. citri* belongs to the same *Xanthomonadaceae* family as *S. maltophilia* and possesses a T6SS that contributes to resistance against eukaryotic predators (54). We discovered that 61 *S*. *maltophilia* complex isolates from animal, human, plant, and environmental sources encode at least one copy of the TssC protein (Fig. 1A; Table S1). Strains harboring TssC-encoding genes are dispersed across multiple countries from Africa, Asia, Australia, Europe, and America, and some were obtained from patient lung, blood, urine, and wound samples (Fig. 1B; Table S1). Three CF isolates (B4, B5, and H_59_creteil) possess TssC-encoding genes (Fig. 1A; Table S1).

Based on their amino acid sequence, *S. maltophilia* complex TssC proteins cluster into three distinct phylogenetic groups: group 1, group 2, or group 3 (Fig. 1A). Group 1 TssC proteins are the most common and share homology to the TssC of *B. cenocepacia* and *Acinetobacter baumannii*, whose TssC sequences belong to the i4b subtype (34, 36, 55, 56). By contrast, TssC proteins from group 2 cluster with TssC1 from *P. aeruginosa*, which belongs to the i3 T6SS subtype, and TssC from *X. citri* (26, 37, 54). Four *S*. *maltophilia* isolates harbor only a single TssC from group 2 and no TssC from group 1 (Fig. 1; Table S2). Finally, *S. maltophilia* complex TssC proteins from group 3 are clustered with TssC2 from *P. aeruginosa* and TssC from *Vibrio cholerae*, which are part of the i1 subtype (34).

**Fig 2:**
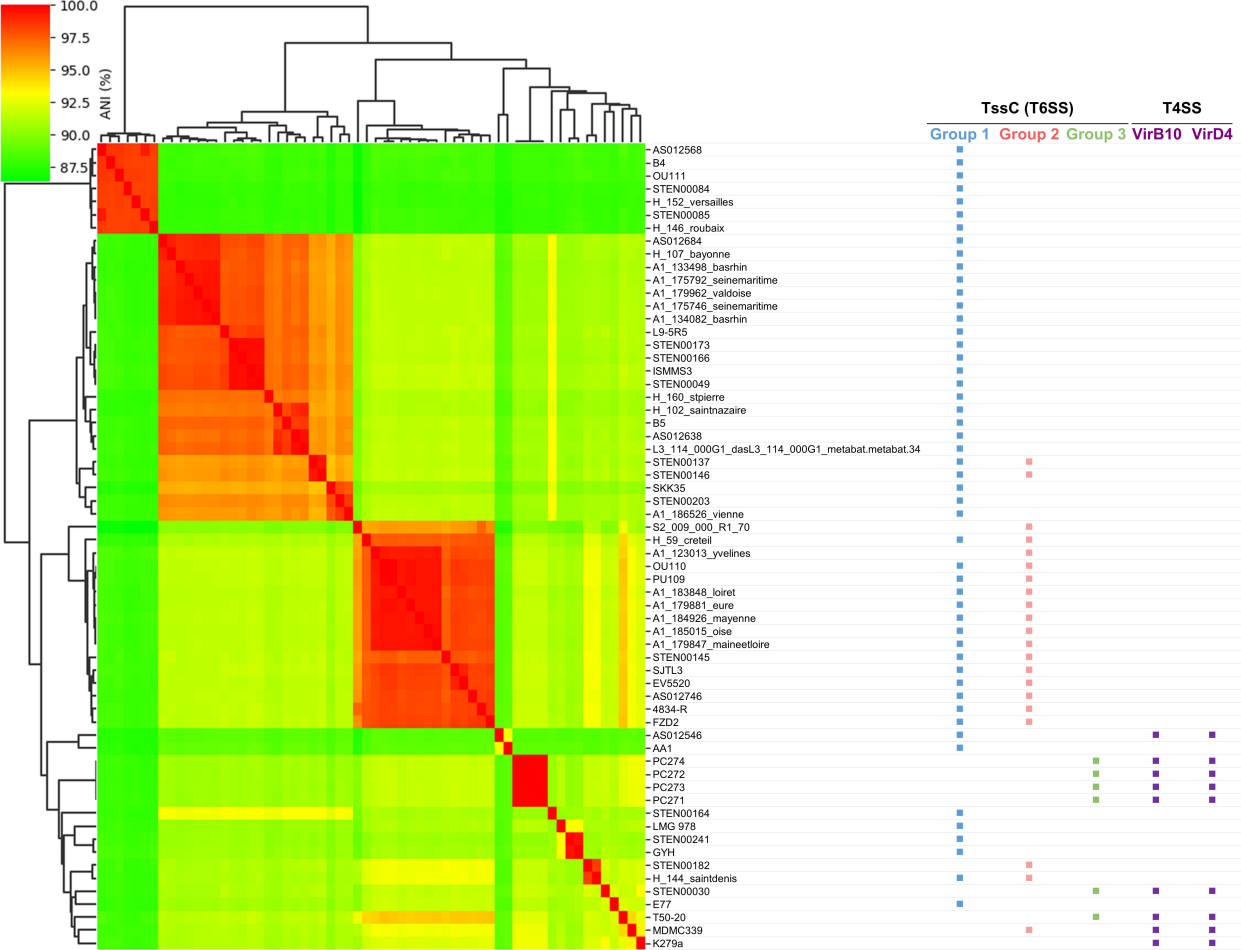
ANI between 61 *S*. *maltophilia* complex strains (and *S. maltophilia* K279a) encoding TssC, VirB10, and VirD4 proteins. Genomic nucleotide sequences of the indicated *S. maltophilia* complex isolates were compared using FastANI, and a matrix was created from the obtained values. Strains were considered to encode VirB10 and VirD4 T4SS proteins (which are essential for the antibacterial activity of the T4SS) if they shared >50% similarity to their respective homologs from K279a. TssC groups are defined in Fig. 1.

We surveyed across *S. maltophilia* complex strains that encode TssC proteins to determine if they also encode VirB10 and VirD4 proteins, which are essential for the antibacterial T4SS in *S. maltophilia* K279a. All *S. maltophilia* complex isolates that encode a TssC protein from group 3, as well as isolate MDMC339 (which encodes a TssC from group 2) and isolate AS012546 (which encodes a TssC from group 1), also encode proteins with 50% or greater homology to the K279a VirB10 and VirD4 proteins (Fig. 2). We next performed an average nucleotide identity (ANI) analysis using *S. maltophilia* complex genomes that encode T6SS proteins. We also included the K279a genome (which encodes T4SS, but not T6SS, proteins) in this ANI analysis (Fig. 2). Most genomes that encode both TssC and Vir proteins cluster near K279a, while many isolates encoding TssC proteins from groups 1 and 2 form a separate group (Fig. 2). In conclusion, *S. maltophilia* complex isolates from both clinical and environmental sources encode diverse TssC proteins that cluster into three distinct groups.

### STEN00241 possesses essential T6SS-encoding genes and a repertoire of predicted toxins

**Fig 3:**
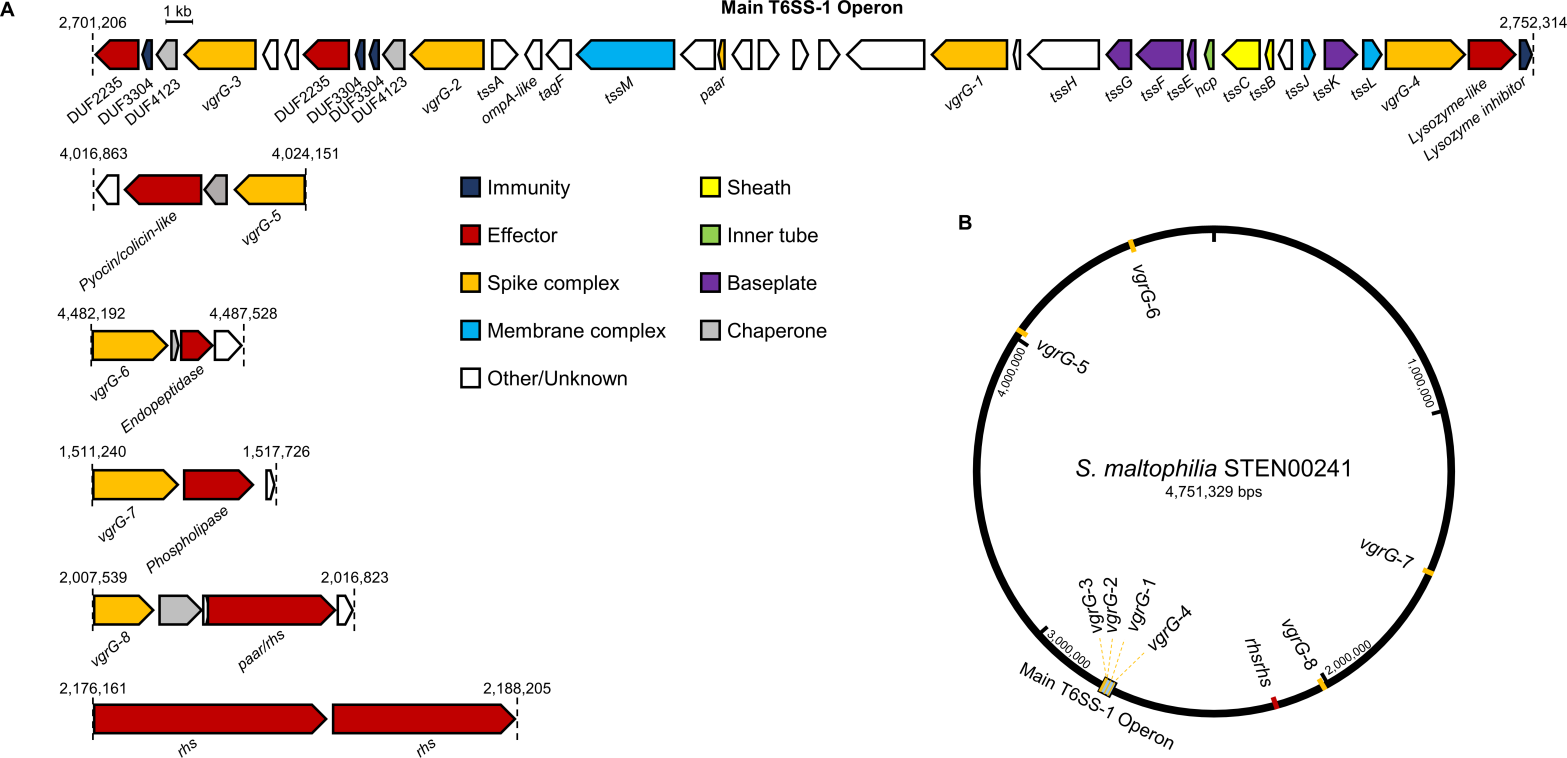
Genes encoding T6SS structural components and toxins are found in *S. maltophilia* complex STEN00241. (**A**) The T6SS-1 cluster of STEN00241 contains T6SS-encoding genes predicted to encode essential components of the membrane complex (blue), baseplate (purple), inner tube (green), outer sheath (yellow), and tip complex (orange). Orphan *vgrG* genes are found near putative toxin and immunity genes. (**B**) Genomic locations of the main T6SS operon and *vgrG* orphan clusters were mapped on the genome of STEN00241.

To further understand the organization and function of T6SS-encoding genes in *S. maltophilia* complex, we analyzed sputum isolate STEN00241, which harbors a single TssC from group 1. STEN00241 possesses a main operon with T6SS-encoding genes (referred to as T6SS-1) predicted to encode proteins that form the membrane complex, baseplate, inner tube, sheath, and the tip of the apparatus (Fig. 3A). Additionally, we discovered eight genes encoding VgrG proteins distributed throughout the genome (Fig. 3A and B). *vgrG-*1, *vgr*G-2, *vgrG-*3, and *vgrG*-4 are found within the main T6SS operon (Fig. 3A). Both *vgrG-*2 and *vgrG-*3 are near genes predicted to encode chaperones (DUF4123), phospholipases (with a DUF2235), and immunity proteins (DUF3304) (46, 57, 58). *vgrG*-4 is located near a gene predicted to encode a protein with a lysozyme-like function.

Unlike *vgrG*1-4, other *vgrG* genes are found at locations distal to the main T6SS operon (Fig. 3B). Downstream of *vgrg-*5*,* a gene encoding a colicin/pyocin-like protein is found, while a putative endopeptidase is encoded downstream of *vgrG-*6. Similarly, a gene predicted to encode a phospholipase is located downstream of *vgrG-*7. Three genes encoding Rhs proteins are also found in the genome of STEN00241: one is located downstream of *vgrG-*8*,* while the other two are found near each other but distant from a *vgrG* gene. These findings demonstrate that *S. maltophilia* complex STEN00241 harbors essential T6SS-encoding genes as well as a diverse array of putative effector-encoding genes.

### The STEN00241 T6SS is active and displays antibacterial properties

**Fig 4:**
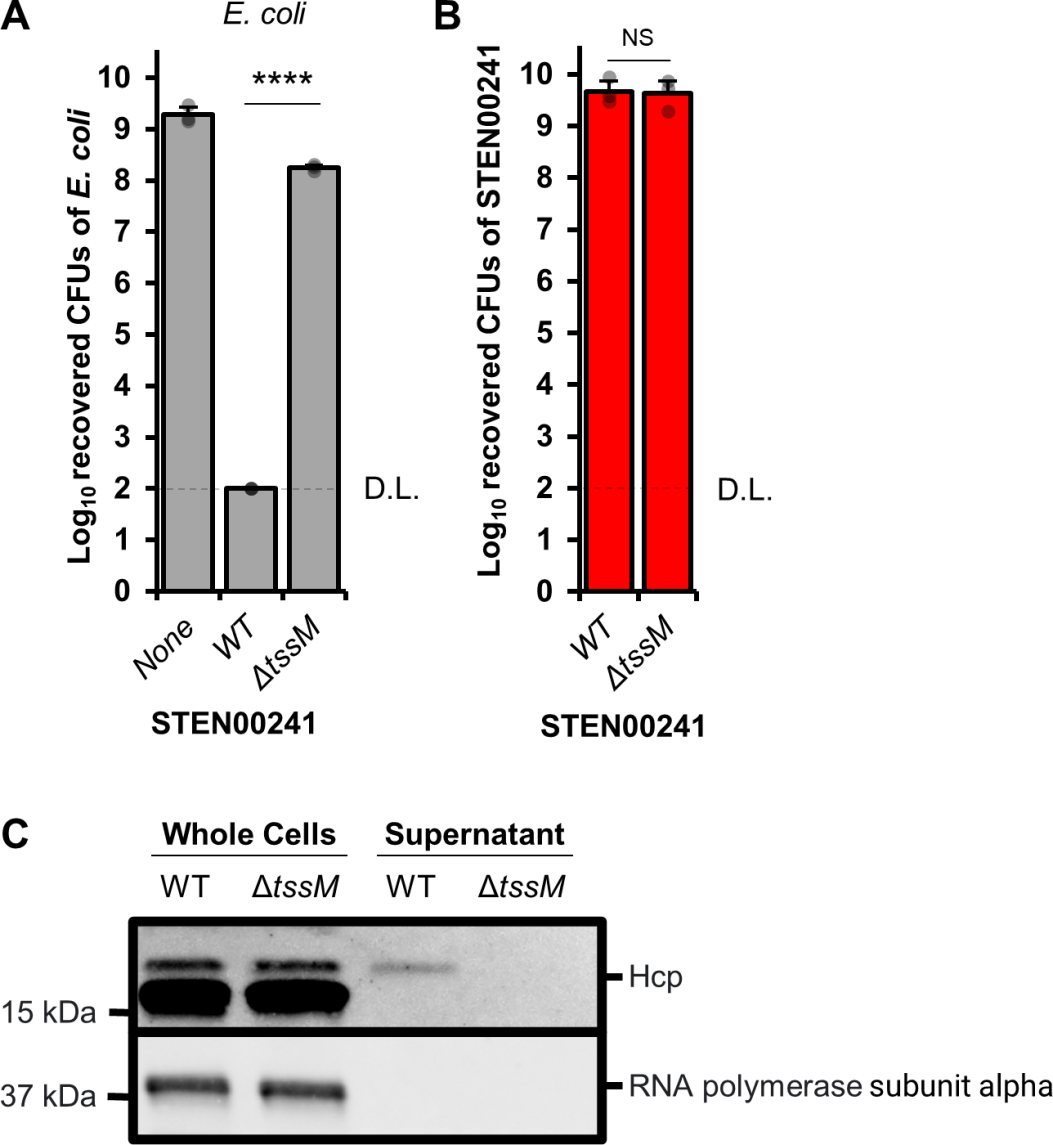
Deletion of the *tssM* gene in STEN00241 abolishes the killing of *E. coli* and secretion of Hcp. (**A**) *E. coli* resistant to tetracycline was grown alone or in the presence of STEN00241 (WT or Δ*tssM*) on solid LB medium at a 10:1 (STEN00241:*E. coli*) ratio for 20 hours at 37°C. The number of surviving *E. coli* cells was determined by plating mixtures on tetracycline plates. (**B**) Same as in panel A, but the number of surviving STEN00241 cells was determined by plating mixtures on imipenem plates. Three independent biological replicates were performed. For panel A, a one-way ANOVA with post-hoc Tukey honestly significant difference (HSD) was used to determine statistical significance. For panel B, Welch’s unequal variances *t*-test was used to determine statistical significance. ****P < 0.0001; NS, not significant (*P* > 0.05). D.L., detection limit. (**C**) Whole cells and supernatants from STEN00241 WT and Δ*tssM* were probed with antibodies against Hcp and RNA polymerase subunit alpha (cell lysis control). Some non-specific bands, which are likely due to the cross-reactivity of the Hcp antibody with other cytoplasmic proteins, were observed for cellular fractions.

Since we observed multiple encoded toxins with putative antibacterial properties in the genome of STEN00241, we hypothesized that the T6SS is used to eliminate competitor bacteria. We engineered a T6SS deficient mutant of STEN00241 by deleting the *tssM* gene (Δ*tssM*), which is essential for the function of T6SS in other bacteria (55, 59). The Δ*tssM* strain has a similar growth rate to the wild type (WT) strain in liquid LB medium (Fig. S1). We co-cultured WT or Δ*tssM* STEN00241 with *E. coli* cells and observed that the WT strain robustly eliminates *E. coli* at 37°C (Fig. 4A) and 25°C (Fig. S2). By contrast, the Δ*tssM* mutant is significantly impaired at killing *E. coli* (Fig. 4A; Fig. S2). Approximately the same number of WT and Δ*tssM* STEN00241 cells are recovered following co-culture with *E. coli* (Fig. 4B). Secretion of the Hcp protein in the supernatant has been previously used to demonstrate active T6SS in other bacterial species (55, 60). We observed that WT *S. maltophilia* secretes Hcp (~18 kDa) in the supernatant, while the Δ*tssM* mutant does not (Fig. 4C).

**Fig 5:**
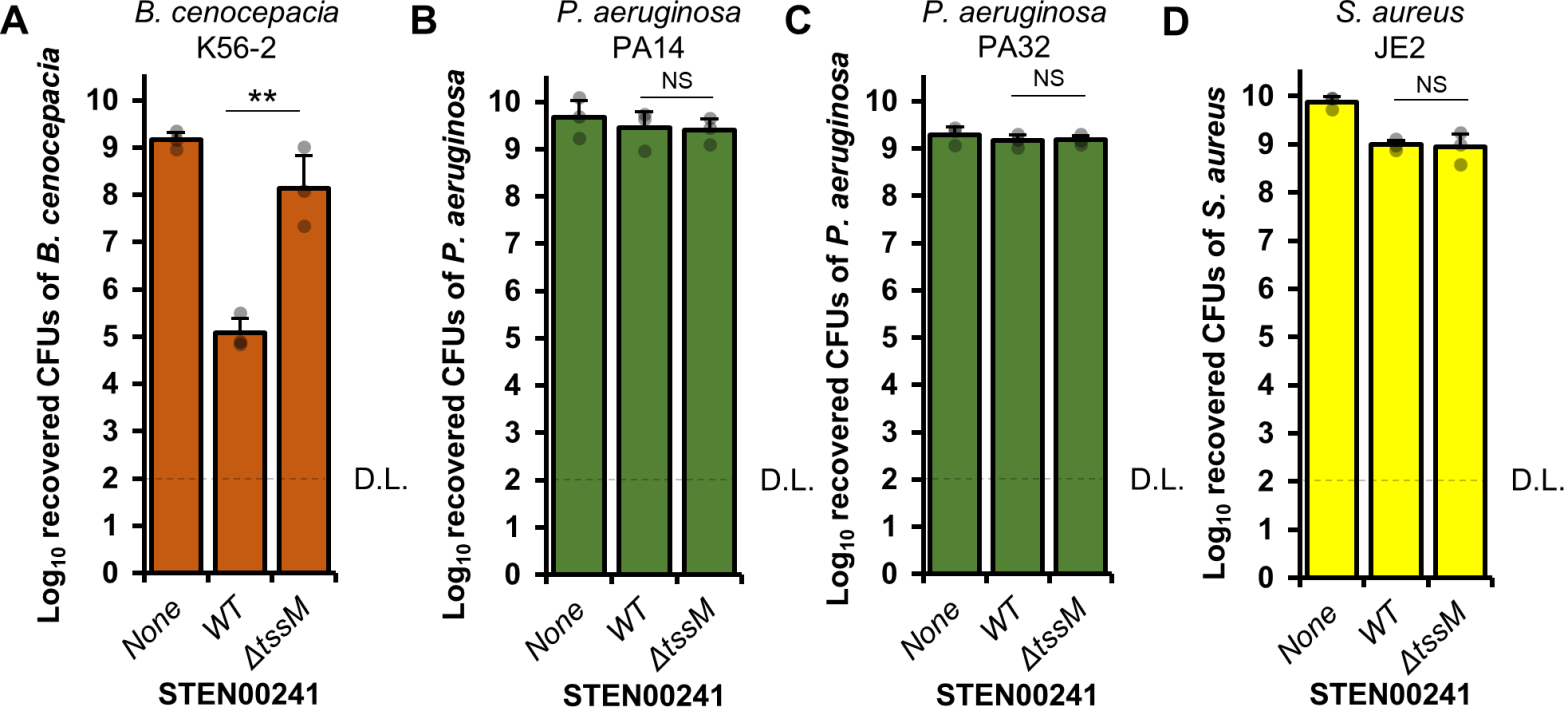
The STEN00241 T6SS contributes to the elimination of *B. cenocepacia,* but not *P. aeruginosa* PA14, PA32, or *S. aureus*. STEN00241 was mixed with target cells at a ratio of 10:1 (STEN00241:target cells) on solid LB medium and incubated for 20 hours at 37°C. (**A**) The number of surviving *B. cenocepacia* target cells was determined by plating mixtures on gentamicin plates. (**B**) The number of surviving *P. aeruginosa* PA14 target cells was determined by plating mixtures on chloramphenicol plates. (**C**) The number of surviving *P. aeruginosa* PA32 target cells was determined by plating mixtures on chloramphenicol plates. (**D**) The number of surviving *S. aureus* target cells was determined by plating mixtures on *Staphylococcus* isolation agar. Three independent biological replicates were performed. A one-way ANOVA with post-hoc Tukey HSD was used to determine statistical significance. **P < 0.01; NS, not significant (*P* > 0.05). D.L., detection limit.

STEN00241 also eliminates *B. cenocepacia* strain K56-2 in a T6SS-dependent manner (Fig. 5A). By contrast, the T6SS does not contribute to killing of the *P. aeruginosa* PA14 laboratory strain or a *P. aeruginosa* CF isolate (PA32; Fig. 5B and C). Similarly, the T6SS does not affect the ability of STEN00241 to eliminate the *Staphylococcus aureus* JE2 laboratory strain (Fig. 5D). These results demonstrate that STEN00241 can utilize the T6SS to eliminate some heterologous bacterial species.

### The T6SS contributes to the competitive fitness of STEN00241 against a co-infecting *P. aeruginosa* isolate

**Fig 6:**
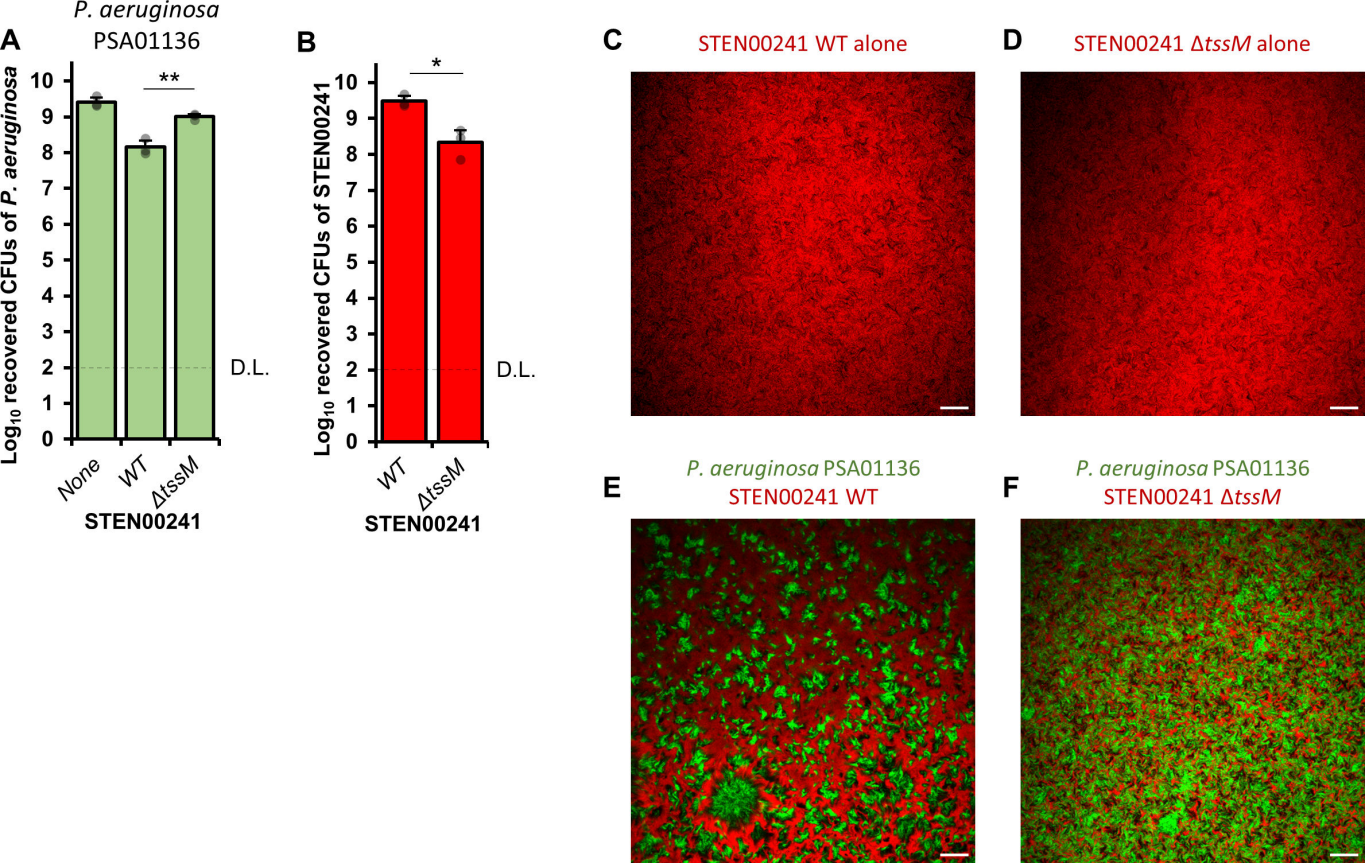
STEN00241 uses the T6SS to compete against a *P. aeruginosa* co-infecting isolate. (**A**) *P. aeruginosa* PSA01136 was grown alone or in the presence of STEN00241 (WT or Δ*tssM*) at a 10:1 (STEN00241:PSA01136) ratio on solid LB medium and incubated for 20 hours at 37°C. The number of surviving *P. aeruginosa* PSA01136 cells was determined by plating mixtures on chloramphenicol plates. (**B**) Same as above, but the number of surviving STEN00241 cells was determined by plating mixtures on imipenem plates. Three independent biological replicates were performed. For panel A, a one-way ANOVA with post-hoc Tukey HSD was used to determine statistical significance. For panel B, Welch’s unequal variances *t*-test was used to determine statistical significance. **P < 0.01, *P < 0.05, NS, not significant (*P* > 0.05). D.L., detection limit. For panels C to F, STEN00241 WT alone expressing mCherry (**C**), STEN00241 Δ*tssM* alone expressing mCherry (**D**), co-cultures between *P. aeruginosa* PSA01136 expressing green fluorescent protein (GFP) and STEN00241 WT expressing mCherry (**E**), or co-cultures between *P. aeruginosa* PSA01136 expressing GFP and STEN00241 Δ*tssM* expressing mCherry (**F**) spotted onto LB plates were visualized without a cover slip using a Zeiss LSM 710 upright microscope. Images were analyzed in FIJI and are representative of three independent biological replicates. The scale bar represents 100 µm.

Interactions that occur between two bacterial species co-isolated from the same patient can have distinct outcomes compared to interactions that occur between strains obtained from different sources (61, 62). STEN00241 was co-isolated from the same patient as *P. aeruginosa* strain PSA01136 (63). We competed STEN00241 and PSA01136 with one another and observed their dynamics. Approximately 10-fold fewer PSA01136 cells are recovered when the strain is competed against WT STEN00241 compared to the Δ*tssM* mutant (Fig. 6A). By contrast, the number of recovered PSA01136 cells is not significantly affected by the T6SS when co-cultures are performed in liquid conditions, which allow only minimal contact to occur between cells (Fig. S3). Since we observed that the STEN00241 T6SS has a significant impact on the survival of the co-infecting PSA01136 strain, we wondered whether the T6SS also influences the survival of STEN00241 during co-cultures with PSA01136. We observed that the number of recovered STEN00241 Δ*tssM* cells is significantly lower compared to the number of recovered WT *S. maltophilia* cells when co-cultured on a solid medium with PSA01136, suggesting that the T6SS also contributes to the survival of STEN00241 against the co-infecting PSA01136 isolate (Fig. 6B).

To determine the impact of the STEN00241 T6SS on the community structure when mixed with the co-infecting PSA01136 isolate, we used confocal microscopy to visualize co-cultures of STEN00241 (WT or Δ*tssM*) expressing mCherry and PSA01136 expressing green fluorescent protein. When PSA01136 is co-cultured with WT STEN00241, *P. aeruginosa* forms large, distinct clusters from which STEN00241 cells are mostly excluded (Fig. 6E; Fig. S4A). By contrast, when PSA01136 is co-cultured with STEN00241 Δ*tssM*, the two strains form a mixed, interwoven pattern (Fig. 6F; Fig. S4B). Taken together, these results provide evidence that the T6SS contributes to the competitive fitness of STEN00241 when co-cultured with a co-infecting *P. aeruginosa* isolate and alters interactions between the two bacteria.

### T4SS- and T6SS-encoding genes are found at distinct genomic locations in *S. maltophilia* complex strains

**Fig 7:**
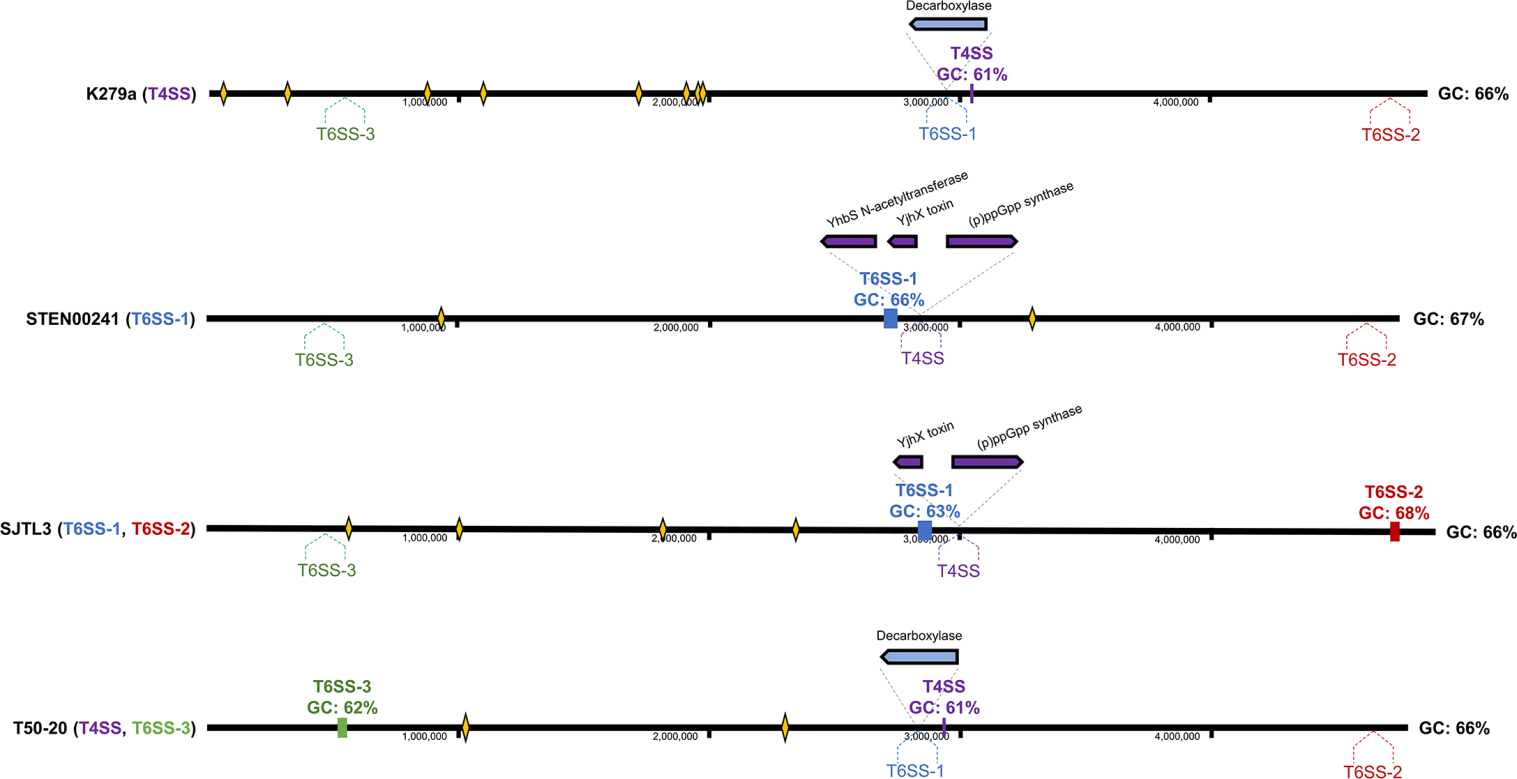
Genes coding for T4SS and T6SS components are found at conserved locations in *S. maltophilia* complex genomes. T4SS (purple), T6SS-1 (blue), T6SS-2 (red), and T6SS-3 (green) genomic sequences were mapped on the genomes of *S. maltophilia* K279a, STEN00241, SJTL3, and T50-20. When sequences were missing, upstream and downstream genomic regions were mapped instead. Dashed lines and names below the solid black lines indicate the putative locations of elements that are missing from the genomes. Rectangles on the black line, gene representations, and names above the solid black line represent elements present in the genomes. Numbers below the lines correspond to genomic locations indicated by the black tick marks in each genome. GC percentages for each gene cluster are displayed below their respective names, and whole genome GC percentages are displayed to the right of each genome. Yellow diamonds represent putative phage elements predicted by PHASTER (64).

Previous work showed that *S. maltophilia* K279a uses a T4SS to eliminate bacteria (20, 21, 24), and here, we provide evidence that the T6SS can also display antibacterial properties. We wondered whether the two systems are found at the same genomic locations in different strains. We analyzed the complete genomes of four *S*. *maltophilia* isolates to compare the genomic locations of T6SS and T4SS gene clusters. For this comparison, we used strain STEN00241, which encodes a TssC from group 1 (within the T6SS-1 cluster), strain K279a, which possesses a T4SS gene cluster but no T6SS-encoding genes, strain SJTL3, which encodes both a TssC from group 1 (within a T6SS-1 cluster) and a TssC from group 2 (within a T6SS-2 cluster), and strain T50-20, which encodes a TssC from group 3 (within a T6SS-3 cluster) and a T4SS (Fig. 7).

From this analysis, we found that the *S. maltophilia* isolates that lack genes encoding T6SS or T4SS elements possess upstream and downstream regions of the missing sequences at approximately the same genomic locations as strains that harbor T6SS or T4SS genes (Fig. 7). SJTL3 and STEN00241 appear to have genes encoding a putative (p)ppGpp synthase/hydrolase and a toxin from a toxin-antitoxin system, where T4SS genes are found in K279a (Fig. 7). Both T50-20 and K279a encode a putative decarboxylase in place of the T6SS-1 cluster. The GC content of the T4SS and T6SS-3 clusters is 61%–62% GC compared to the 66% overall genomic GC content of K279a and T50-20. We did not detect phage-related sequences in the immediate vicinity (~50,000 nucleotides) of T4SS or T6SS modules (Fig. 7, yellow diamonds).

## DISCUSSION

The study presented here describes the T6SS as an antibacterial weapon in the arsenal of *S. maltophilia* complex. In both host and environmental settings, bacteria like *S. maltophilia* live in polymicrobial communities, where interactions with other cells influence the ability of a species to persist (65). These interactions are often antagonistic and may be mediated by diffusible antibacterial small molecules or by contact-dependent proteins that are transferred between cells to intoxicate competitors (25, 65).

We report here that T6SS-encoding genes are found in geographically diverse *S. maltophilia* complex isolates obtained from patient and non-patient sources, suggesting that the T6SS might confer a competitive advantage against other bacteria during infection as well as for survival in the environment. This hypothesis is supported by our finding that the T6SS is important for the elimination of *E. coli* at both 25°C and 37°C. *P. aeruginosa* and *B. cenocepacia* also possess active T6SS clusters that may contribute to pathogenicity and allow those pathogens to outcompete other bacteria (25, 27). *P. aeruginosa* Hcp proteins and antibodies against Hcp have been detected in the sputum of CF patients (26). *P. aeruginosa* CF isolates with loss-of-function mutations in T6SS regulator genes become susceptible to killing by species from the *Burkholderia cepacia* complex (27). It is currently unclear if the T6SS of *S. maltophilia* is important in infections such as those seen in CF patients.

*P. aeruginosa* and *S. maltophilia* complex species are frequently isolated from the same patients, and both cooperative and antagonistic interactions between the two pathogens have been described (20–22). During mouse lung infections, *P. aeruginosa* can increase *S. maltophilia* proliferation and associated immune responses (22). Patient isolates of *P. aeruginosa* and *S. maltophilia* have been observed to share genomic sequences, suggesting that inter-species horizontal gene transfer is common among these organisms (66). However, competitive interactions can also occur between these two pathogens. *S. maltophilia* K279a uses the T4SS to deliver toxins with predicted lipase and lysozyme-like activity to intoxicate and kill *P. aeruginosa* strains (20, 21, 24). We provide evidence that an *S. maltophilia* complex pulmonary isolate can utilize the T6SS to eliminate heterologous bacteria like *E. coli* and *B. cenocepacia* and to compete against a co-infecting *P. aeruginosa* isolate.

Even though the T6SS contributes to the competitive fitness of STEN00241 against its co-infecting *P. aeruginosa* PSA01136 strain, it does not play a significant role in competition against the lab strain PA14 or the CF isolate PA32. Perault et al. observed that *P. aeruginosa* isolates from adult patients harbor mutations in the *gacS*, *gacA*, *retS*, *fha1*, and *pppA* genes that encode T6SS regulators in *P. aeruginosa* (27). We also observed mutations in these genes in *P. aeruginosa* PSA01136 when compared to PA14. However, the impact played by those mutations in mediating PSA01136 T6SS activity is unclear. *P. aeruginosa* employs defense mechanisms against T6SS attacks, such as the Arc immunity pathways and stress response systems (66, 67). We suspect that variable expression of defensive and offensive systems in the co-infecting PSA01136 isolate could explain the differences we observed in their competitive fitness against STEN00241 compared to PA14. Although some exceptions have been recently identified, most T6SS are not effective at eliminating Gram-positive bacteria (68). Competition with STEN00241 reduced the number of recovered *S. aureus* during co-cultures compared to monocultures, but the T6SS did not affect the survival of *S. aureus*.

The distribution and organization of T6SS-encoding genes across bacterial genomes are diverse. Species from the *Burkholderia* genus may harbor up to six distinct T6SS clusters, while *P. aeruginosa* strains generally possess three T6SS clusters (26, 69). *V. cholerae* strains employ a single T6SS operon that encodes structural and regulatory proteins, as well as orphan *vgrG* gene loci that contain effectors with diverse functions (70). We found that *S. maltophilia* complex strains can encode TssC proteins from groups 1 and 2, but all isolates that encode a TssC from group 3 only encode a single TssC protein. All *S. maltophilia* complex strains with a TssC from group 3 also harbor genes encoding T4SS essential components similar to the ones used by K279a to eliminate bacteria (20, 21, 24). In other bacteria that harbor multiple T6SS clusters, each system can play a role in mediating virulence, obtaining nutrients, and conferring competitive advantages against other bacteria (71–73). It is unclear why some *S. maltophilia* complex isolates harbor T4SS genes while others possess T6SS components. Based on the four complete genomes analyzed in Fig. 7, T4SS or T6SS modules appear at conserved but distinct genomic locations, suggesting that they are not a part of interchanged mobile elements. We propose that *S. maltophilia* complex strains have separate genomic locations which serve as an armory that accommodates different molecular weapons. We speculate that evolutionary pressures dictated by the environment, like competition with eukaryotic or prokaryotic cells, the energy cost of assembling and firing a T4SS or T6SS in different conditions, and the efficacy of secreted toxins, might play key roles in determining which secretion system is acquired and maintained by a *S. maltophilia* complex strain. Future work will determine whether different *S. maltophilia* T6SS are required for different processes.

Results presented here enhance our understanding of the secretion systems and antibacterial weapons employed by an important emerging pathogen. We propose that strains harboring T6SS-encoding genes have competitive fitness advantages during survival in both infections and on hospital or environmental surfaces. Additional studies will elucidate how the *S. maltophilia* complex T6SS is regulated, which components of the apparatus are required for activity, which toxins are important in mediating the observed competitive fitness advantages, and how the system might affect virulence. Understanding the mechanisms of the antibacterial T6SS in *S. maltophilia* complex could lead to the development of treatment strategies against strains resistant to antibiotics.

## MATERIALS AND METHODS

### Bacterial strains and growth conditions

*S. maltophilia* complex STEN00241 and *P. aeruginosa* strain PSA01136 were previously isolated and sequenced as a part of a bacterial whole genome sequencing surveillance study (63). *E. coli* DH5α, *B. cenocepacia* K56-2, *P. aeruginosa* PA14, and *S. aureus* JE2 were used for co-culture assays with STEN00241. *P. aeruginosa* PA32 (CFBR509_Pae_20170525_S_EBPa32) was isolated from the sputum of a CF patient (61). *E. coli* SM10 was used to conjugate plasmids into *S. maltophilia*. Strains were routinely grown in LB medium at 37*°*C unless otherwise indicated. The following antibiotic concentrations were used where appropriate: chloramphenicol (20 µg/mL), imipenem (20 µg/mL or 32 µg/mL for imipenem monohydrate), gentamicin (75 µg/mL), and tetracycline (20 µg/mL). All strains used in this study are listed in Table S2.

### Construction of phylogenetic trees and ANI matrix

*S. maltophilia* complex protein sequences from both RefSeq and Genbank NCBI databases were retrieved in June 2022 and used to create a local BLAST database. A local BlastP search with a maximum E-value of 0.05 and a BLOSUM62 matrix was conducted using the TssC protein sequence of *X. citri*. Incomplete hits were eliminated from further analyses. *S. maltophilia* complex TssC sequences and TssC sequences from *X. citri*, *P. aeruginosa* PAO1 (TssC1 and TssC2), *V. cholerae* V52, *B. cenocepacia* K56-2, and *A. baumannii* DSM 30011 were aligned using MUSCLE (74). Alignments were used to construct a phylogenetic tree in PhyML with a Bayesian Information Criterion Smart Model Selection (75, 76). Branch supports were calculated using the aLRT SH-like fast likelihood-based method (75). The final tree was created with iTOL (77). *S. maltophilia* genomes encoding a TssC protein were also confirmed to encode TssB and TssM T6SS proteins. The ANI of *S. maltophilia* complex genomes harboring T6SS-encoding genes was calculated using FastANI, and a pairwise ANI matrix was built using ANIClustermap (78).

### Molecular biology

Standard molecular biology techniques were used to construct plasmids and PCR products. All restriction enzymes, DNA polymerases, and Gibson mixes were utilized according to manufacturer instructions. Plasmid constructs and PCR products were sequenced by GENEWIZ (Azenta Life Sciences, Chelmsford, MA, USA).

### Genetic manipulation of *S. maltophilia*

To engineer the *S. maltophilia* complex STEN00241 Δ*tssM* mutant, a similar allelic exchange method to the one described by Welker et al. was used (79). Briefly, 1,000 bp upstream and downstream of the *tssM* gene (including the start and stop codons) were assembled on the pEX18Tc suicide vector using Gibson assembly. The pEX18Tc-Δ*tssM* construct was transformed into *E. coli* SM10 and then conjugated into STEN00241. Conjugants were identified by growth on tetracycline and imipenem and then plated onto freshly made 15% sucrose LB plates (with no NaCl). Colonies were screened by PCR and confirmed by Sanger sequencing. Plasmids used in this study are listed in Table S2, and primers are listed in Table S3.

### Co-culture assays

Overnight cultures of the indicated strains were made from single colonies and were diluted 1:100 in fresh LB medium. Cultures were grown for 4 hours and set to an OD_600_ = 1.0. For *E. coli* DH5α carrying the tetracycline-resistant pEX18Tc plasmid, tetracycline was added to the growth media, and cultures were washed with fresh LB before co-culturing with *S. maltophilia*. A 1× volume of target strains was mixed with a 10× volume *S*. *maltophilia* and centrifuged, and mixtures were resuspended in a 1× volume of LB. 5 µL of the mixed strains or of the indicated strains alone were spotted on LB plates (or added to 3 mL of liquid LB). Co-cultures were then incubated at 37°C (or 25°C where indicated) for 20 hours. For co-cultures spotted on LB plates, colonies were excised, placed in 1 mL of liquid LB, and vortexed for 30 seconds. Cells were then serially diluted and plated on the following selective plates: tetracycline (20 µg/mL) to select for *E. coli*, chloramphenicol (20 µg/mL) to select for *P. aeruginosa*, gentamicin (75 µg/mL) to select for *B. cenocepacia*, *Staphylococcus* isolation agar (Trypticase soy agar with 7.5% NaCl) to select for *S. aureus*, and imipenem to select for *S. maltophilia*.

### Confocal microscopy

Plasmid pMRP9-1 (GFP+) (80) was introduced into *P. aeruginos*a PSA01136, and plasmid pMP7605 (mCherry) (81) was introduced into *S. maltophilia* complex STEN00241 (both WT and Δ*tssM*) by electroporation. STEN00241 WT and Δ*tssM* expressing mCherry and *P. aeruginos*a PSA01136 expressing GFP+ were inoculated into LB medium from single colonies. Overnight cultures were diluted 1:100 in fresh LB medium, grown for 4 hours, washed with LB, and set to an OD_600_ = 1.0. A 1× volume of PSA01136 expressing GFP+ was mixed with a 10× volume STEN00241 mCherry*,* centrifuged, and resuspended in 1× LB volume. Five microliters of the mixed strains or the indicated strains alone was spotted on dry LB plates and grown overnight. Colonies were visualized without a cover slip from the same LB plates on which they were spotted using a Zeiss LSM 710 upright microscope equipped with a Plan-Apochromat 10× objective. A 488 nm laser was used to observe *P. aeruginos*a PSA01136 expressing GFP+, and a 555 nm laser was used to observe *S. maltophilia* expressing mCherry. Images were analyzed in FIJI. Representative images of three biological replicates are shown. Representative fields of view were taken from the colonies of each biological replicate.

### Hcp immunoblotting

Polyclonal rabbit antibodies against the STEN00241 Hcp peptide N-LLQPRSATASTSGG-C were generated by Genescript. Overnight cultures of WT or Δ*tssM* STEN00241 were washed with LB and back-diluted to an OD_600_ = 0.01. Strains were grown at 37°C to an OD_600_ ≈ 0.7. For cell fractions, 300 µL of cultures were centrifuged, and cell pellets were resuspended in 100 µL of 1× Laemmli buffer. Samples were incubated at 99°C for 30 minutes. For supernatant fractions, cultures were centrifuged for 10 minutes at 2,000 × *g*, supernatants were filter sterilized twice using 0.22 µm filters, and 700 µL of the filtered supernatants were incubated with trichloroacetic acid at a final concentration of 20% overnight at 4°C. Samples were then centrifuged for 10 minutes and washed three times with cold acetone. Precipitated proteins were resuspended in 150 µL of 1× Laemmli buffer in sodium dodecyl sulfate (SDS) buffer and incubated at 99°C for 60 minutes. Proteins from both cellular and supernatant fractions were separated on an SDS-PAGE gel and transferred to a polyvinylidene difluoride (PVDF) membrane. The membrane was probed with primary antibodies against Hcp (1:1,000) and RNA polymerase subunit alpha (1:2,000) and secondary antibodies against rabbit (for Hcp) and mouse (for RNA polymerase subunit alpha) (1:5,000, LI-COR Biosciences). The membrane was imaged using a Bio-Rad ChemiDoc imager and analyzed in FIJI.

## SUPPLEMENTAL MATERIAL

Supplemental Figures msphere.00584-23-s0001.docx Figures S1-S4.

Supplemental Tables msphere.00584-23-s0002.docx Tables S1-S3.

ASM does not own the copyrights to Supplemental Material that may be linked to, or accessed through, an article. The authors have granted ASM a non-exclusive, world-wide license to publish the Supplemental Material files. Please contact the corresponding author directly for reuse.

## References

[1] Khanum I, Ilyas A, Ali F. 2020. Stenotrophomonas maltophilia meningitis - a case series and review of the literature. Cureus 12:e11221. doi:10.7759/cureus.11221 33269149 PMC7704165

[2] Kaito S, Sekiya N, Najima Y, Sano N, Horiguchi S, Kakihana K, Hishima T, Ohashi K. 2018. Fatal neutropenic enterocolitis caused by Stenotrophomonas maltophilia: a rare and underrecognized entity. Intern Med 57:3667–3671. doi:10.2169/internalmedicine.1227-18 30101922 PMC6355424

[3] Rémi J, Loesch-Biffar AM, Mehrkens J, Thon N, Seelos K, Pfister H-W. 2019. Stenotrophomonas maltophilia brain abscesses after implantation of motor cortex stimulator. J Neurol Sci 400:32–33. doi:10.1016/j.jns.2019.03.010 30889467

[4] Kim EJ, Kim YC, Ahn JY, Jeong SJ, Ku NS, Choi JY, Yeom J-S, Song YG. 2019. Risk factors for mortality in patients with Stenotrophomonas maltophilia bacteremia and clinical impact of quinolone-resistant strains. BMC Infect Dis 19:754. doi:10.1186/s12879-019-4394-4 31462215 PMC6714101

[5] Sánchez MB. 2015. Antibiotic resistance in the opportunistic pathogen Stenotrophomonas maltophilia. Front Microbiol 6:658. doi:10.3389/fmicb.2015.00658 26175724 PMC4485184

[6] Brooke JS. 2021. Advances in the microbiology of Stenotrophomonas maltophilia. Clin Microbiol Rev 34:e0003019. doi:10.1128/CMR.00030-19 34043457 PMC8262804

[7] Kumar S, Bansal K, Patil PP, Kaur A, Kaur S, Jaswal V, Gautam V, Patil PB. 2020. Genomic insights into evolution of extensive drug resistance in Stenotrophomonas maltophilia complex. Genomics 112:4171–4178. doi:10.1016/j.ygeno.2020.06.049 32653516

[8] Cai B, Tillotson G, Benjumea D, Callahan P, Echols R. 2020. The burden of bloodstream infections due to Stenotrophomonas maltophilia in the United States: a large, retrospective database study. Open Forum Infect Dis 7:ofaa141. doi:10.1093/ofid/ofaa141 32462047 PMC7240339

[9] Kim S-H, Cho SY, Kang C-I, Seok H, Huh K, Ha YE, Chung DR, Lee NY, Peck KR, Song J-H. 2018. Clinical predictors of Stenotrophomonas maltophilia bacteremia in adult patients with hematologic malignancy. Ann Hematol 97:343–350. doi:10.1007/s00277-017-3178-4 29138886

[10] Guo J, Garratt A, Hill A. 2022. Worldwide rates of diagnosis and effective treatment for cystic fibrosis. J Cyst Fibros 21:456–462. doi:10.1016/j.jcf.2022.01.009 35125294

[11] Cuthbertson L, Walker AW, Oliver AE, Rogers GB, Rivett DW, Hampton TH, Ashare A, Elborn JS, De Soyza A, Carroll MP, Hoffman LR, Lanyon C, Moskowitz SM, O’Toole GA, Parkhill J, Planet PJ, Teneback CC, Tunney MM, Zuckerman JB, Bruce KD, van der Gast CJ. 2020. Lung function and microbiota diversity in cystic fibrosis. Microbiome 8:45. doi:10.1186/s40168-020-00810-3 32238195 PMC7114784

[12] Waters V, Yau Y, Prasad S, Lu A, Atenafu E, Crandall I, Tom S, Tullis E, Ratjen F. 2011. Stenotrophomonas maltophilia in cystic fibrosis. Am J Respir Crit Care Med 183:635–640. doi:10.1164/rccm.201009-1392OC 20889901

[13] Stanojevic S, Ratjen F, Stephens D, Lu A, Yau Y, Tullis E, Waters V. 2013. Factors influencing the acquisition of Stenotrophomonas maltophilia infection in cystic fibrosis patients. J Cyst Fibros 12:575–583. doi:10.1016/j.jcf.2013.05.009 23757360

[14] Yang S, Hua M, Liu X, Du C, Pu L, Xiang P, Wang L, Liu J. 2021. Bacterial and fungal co-infections among COVID-19 patients in intensive care unit. Microbes Infect 23:104806. doi:10.1016/j.micinf.2021.104806 33684520 PMC7933791

[15] Ishikawa K, Nakamura T, Kawai F, Uehara Y, Mori N. 2022. Stenotrophomonas maltophilia infection associated with COVID-19: a case series and literature review. Am J Case Rep 23:e936889. doi:10.12659/AJCR.936889 35852985 PMC9308482

[16] Langford BJ, So M, Simeonova M, Leung V, Lo J, Kan T, Raybardhan S, Sapin ME, Mponponsuo K, Farrell A, Leung E, Soucy J-P, Cassini A, MacFadden D, Daneman N, Bertagnolio S. 2023. Antimicrobial resistance in patients with COVID-19: a systematic review and meta-analysis. Lancet Microbe 4:e179–e191. doi:10.1016/S2666-5247(22)00355-X 36736332 PMC9889096

[17] Kusaba T, Kirita Y, Ishida R, Matsuoka E, Nakayama M, Uchiyama H, Kajita Y. 2012. Morphological analysis of biofilm of peritoneal dialysis catheter in refractory peritonitis patient. CEN Case Rep 1:50–54. doi:10.1007/s13730-012-0012-7 28509153 PMC5413638

[18] Liu B, Tong S. 2019. An investigation of Stenotrophomonas maltophilia-positive culture caused by fiberoptic bronchoscope contamination. BMC Infect Dis 19:1072. doi:10.1186/s12879-019-4670-3 31864284 PMC6925470

[19] Gröschel MI, Meehan CJ, Barilar I, Diricks M, Gonzaga A, Steglich M, Conchillo-Solé O, Scherer I-C, Mamat U, Luz CF, et al.. 2020. The phylogenetic landscape and nosocomial spread of the multidrug-resistant opportunist Stenotrophomonas maltophilia. Nat Commun 11:2044. doi:10.1038/s41467-020-15123-0 32341346 PMC7184733

[20] Bayer-Santos E, Cenens W, Matsuyama BY, Oka GU, Di Sessa G, Mininel IDV, Alves TL, Farah CS. 2019. The opportunistic pathogen Stenotrophomonas maltophilia utilizes a type IV secretion system for interbacterial killing. PLoS Pathog 15:e1007651. doi:10.1371/journal.ppat.1007651 31513674 PMC6759196

[21] Nas MY, White RC, DuMont AL, Lopez AE, Cianciotto NP. 2019. Stenotrophomonas maltophilia encodes a VirB/VirD4 type IV secretion system that modulates apoptosis in human cells and promotes competition against heterologous bacteria, including Pseudomonas aeruginosa. Infect Immun 87:e00457-19. doi:10.1128/IAI.00457-19 31235638 PMC6704607

[22] McDaniel MS, Schoeb T, Swords WE. 2020. Cooperativity between Stenotrophomonas maltophilia and Pseudomonas aeruginosa during polymicrobial airway infections. Infect Immun 88:e00855-19. doi:10.1128/IAI.00855-19 31932329 PMC7093137

[23] Pompilio A, Crocetta V, De Nicola S, Verginelli F, Fiscarelli E, Di Bonaventura G. 2015. Cooperative pathogenicity in cystic fibrosis: Stenotrophomonas maltophilia modulates Pseudomonas aeruginosa virulence in mixed biofilm. Front Microbiol 6:951. doi:10.3389/fmicb.2015.00951 26441885 PMC4584994

[24] Nas MY, Gabell J, Cianciotto NP. 2021. Effectors of the Stenotrophomonas maltophilia type IV secretion system mediate killing of clinical isolates of Pseudomonas aeruginosa. mBio 12:e0150221. doi:10.1128/mBio.01502-21 34182776 PMC8262851

[25] Crisan CV, Goldberg JB. 2022. Antibacterial contact-dependent proteins secreted by Gram-negative cystic fibrosis respiratory pathogens. Trends Microbiol 30:986–996. doi:10.1016/j.tim.2022.03.009 35487848 PMC9474641

[26] Mougous JD, Cuff ME, Raunser S, Shen A, Zhou M, Gifford CA, Goodman AL, Joachimiak G, Ordoñez CL, Lory S, Walz T, Joachimiak A, Mekalanos JJ. 2006. A virulence locus of Pseudomonas aeruginosa encodes a protein secretion apparatus. Science 312:1526–1530. doi:10.1126/science.1128393 16763151 PMC2800167

[27] Perault AI, Chandler CE, Rasko DA, Ernst RK, Wolfgang MC, Cotter PA. 2020. Host adaptation predisposes Pseudomonas aeruginosa to type VI secretion system-mediated predation by the Burkholderia cepacia complex. Cell Host Microbe 28:534–547. doi:10.1016/j.chom.2020.06.019 32755549 PMC7554260

[28] Brunet YR, Zoued A, Boyer F, Douzi B, Cascales E, Viollier PH. 2015. The type VI secretion TssEFGK-VgrG phage-like baseplate is recruited to the TssJLM membrane complex via multiple contacts and serves as assembly platform for tail tube/sheath polymerization. PLoS Genet 11:e1005545. doi:10.1371/journal.pgen.1005545 26460929 PMC4604203

[29] Durand E, Nguyen VS, Zoued A, Logger L, Péhau-Arnaudet G, Aschtgen M-S, Spinelli S, Desmyter A, Bardiaux B, Dujeancourt A, Roussel A, Cambillau C, Cascales E, Fronzes R. 2015. Biogenesis and structure of a type VI secretion membrane core complex. Nature 523:555–560. doi:10.1038/nature14667 26200339

[30] Chang Y-W, Rettberg LA, Ortega DR, Jensen GJ. 2017. In vivo structures of an intact type VI secretion system revealed by electron cryotomography. EMBO Rep 18:1090–1099. doi:10.15252/embr.201744072 28487352 PMC5494534

[31] Vettiger A, Basler M. 2016. Type VI secretion system substrates are transferred and reused among sister cells. Cell 167:99–110. doi:10.1016/j.cell.2016.08.023 27616061

[32] Kube S, Kapitein N, Zimniak T, Herzog F, Mogk A, Wendler P. 2014. Structure of the VipA/B type VI secretion complex suggests a contraction-state-specific recycling mechanism. Cell Rep 8:20–30. doi:10.1016/j.celrep.2014.05.034 24953649

[33] Russell AB, Wexler AG, Harding BN, Whitney JC, Bohn AJ, Goo YA, Tran BQ, Barry NA, Zheng H, Peterson SB, Chou S, Gonen T, Goodlett DR, Goodman AL, Mougous JD. 2014. A type VI secretion-related pathway in Bacteroidetes mediates interbacterial antagonism. Cell Host Microbe 16:227–236. doi:10.1016/j.chom.2014.07.007 25070807 PMC4136423

[34] Li J, Yao Y, Xu HH, Hao L, Deng Z, Rajakumar K, Ou H-Y. 2015. SecReT6: a web-based resource for type VI secretion systems found in bacteria. Environ Microbiol 17:2196–2202. doi:10.1111/1462-2920.12794 25640659

[35] Cherrak Y, Flaugnatti N, Durand E, Journet L, Cascales E. 2019. Structure and activity of the type VI secretion system. Microbiol Spectr 7. doi:10.1128/microbiolspec.PSIB-0031-2019 PMC10957189 31298206

[36] Boyer F, Fichant G, Berthod J, Vandenbrouck Y, Attree I. 2009. Dissecting the bacterial type VI secretion system by a genome wide in silico analysis: what can be learned from available microbial genomic resources. BMC Genomics 10:104. doi:10.1186/1471-2164-10-104 19284603 PMC2660368

[37] Barret M, Egan F, Fargier E, Morrissey JP, O’Gara F. 2011. Genomic analysis of the type VI secretion systems in Pseudomonas spp.: novel clusters and putative effectors uncovered. Microbiology (Reading) 157:1726–1739. doi:10.1099/mic.0.048645-0 21474537

[38] Wood TE, Howard SA, Wettstadt S, Filloux A. 2019. PAAR proteins act as the ‘sorting hat’ of the type VI secretion system. Microbiology (Reading) 165:1203–1218. doi:10.1099/mic.0.000842 31380737 PMC7376260

[39] Pissaridou P, Allsopp LP, Wettstadt S, Howard SA, Mavridou DAI, Filloux A. 2018. The Pseudomonas aeruginosa T6SS-VgrG1b spike is topped by a PAAR protein eliciting DNA damage to bacterial competitors. Proc Natl Acad Sci U S A 115:12519–12524. doi:10.1073/pnas.1814181115 30455305 PMC6298103

[40] Bondage DD, Lin J-S, Ma L-S, Kuo C-H, Lai E-M. 2016. VgrG C terminus confers the type VI effector transport specificity and is required for binding with PAAR and adaptor–effector complex. Proc Natl Acad Sci U S A 113:E3931–E3940. doi:10.1073/pnas.1600428113 27313214 PMC4941472

[41] Monjarás Feria J, Valvano MA. 2020. An overview of anti-eukaryotic T6SS effectors. Front Cell Infect Microbiol 10:584751. doi:10.3389/fcimb.2020.584751 33194822 PMC7641602

[42] Burkinshaw BJ, Liang X, Wong M, Le ANH, Lam L, Dong TG. 2018. A type VI secretion system effector delivery mechanism dependent on PAAR and a chaperone–co-chaperone complex. Nat Microbiol 3:632–640. doi:10.1038/s41564-018-0144-4 29632369

[43] Günther P, Quentin D, Ahmad S, Sachar K, Gatsogiannis C, Whitney JC, Raunser S, Satchell KJF. 2022. Structure of a bacterial Rhs effector exported by the type VI secretion system. PLoS Pathog 18:e1010182. doi:10.1371/journal.ppat.1010182 34986192 PMC8765631

[44] Donato SL, Beck CM, Garza-Sánchez F, Jensen SJ, Ruhe ZC, Cunningham DA, Singleton I, Low DA, Hayes CS. 2020. The β-encapsulation cage of rearrangement hotspot (Rhs) effectors is required for type VI secretion. Proc Natl Acad Sci U S A 117:33540–33548. doi:10.1073/pnas.1919350117 33323487 PMC7777165

[45] Russell AB, Hood RD, Bui NK, LeRoux M, Vollmer W, Mougous JD. 2011. Type VI secretion delivers bacteriolytic effectors to target cells. Nature 475:343–347. doi:10.1038/nature10244 21776080 PMC3146020

[46] Russell AB, LeRoux M, Hathazi K, Agnello DM, Ishikawa T, Wiggins PA, Wai SN, Mougous JD. 2013. Diverse type VI secretion phospholipases are functionally plastic antibacterial effectors. Nature 496:508–512. doi:10.1038/nature12074 23552891 PMC3652678

[47] Mariano G, Trunk K, Williams DJ, Monlezun L, Strahl H, Pitt SJ, Coulthurst SJ. 2019. A family of type VI secretion system effector proteins that form ion-selective pores. Nat Commun 10:5484. doi:10.1038/s41467-019-13439-0 31792213 PMC6889166

[48] Ahmad S, Wang B, Walker MD, Tran HKR, Stogios PJ, Savchenko A, Grant RA, McArthur AG, Laub MT, Whitney JC. 2019. An interbacterial toxin inhibits target cell growth by synthesizing (p)ppApp. Nature 575:674–678. doi:10.1038/s41586-019-1735-9 31695193 PMC6883173

[49] Whitney JC, Quentin D, Sawai S, LeRoux M, Harding BN, Ledvina HE, Tran BQ, Robinson H, Goo YA, Goodlett DR, Raunser S, Mougous JD. 2015. An interbacterial NAD(P)+ glycohydrolase toxin requires elongation factor Tu for delivery to target cells. Cell 163:607–619. doi:10.1016/j.cell.2015.09.027 26456113 PMC4624332

[50] Nolan LM, Cain AK, Clamens T, Furniss RCD, Manoli E, Sainz-Polo MA, Dougan G, Albesa-Jové D, Parkhill J, Mavridou DAI, Filloux A. 2021. Identification of Tse8 as a type VI secretion system toxin from Pseudomonas aeruginosa that targets the bacterial transamidosome to inhibit protein synthesis in prey cells. Nat Microbiol 6:1199–1210. doi:10.1038/s41564-021-00950-8 34413503 PMC7611593

[51] Crisan CV, Chandrashekar H, Everly C, Steinbach G, Hill SE, Yunker PJ, Lieberman RR, Hammer BK. 2021. A new contact killing toxin permeabilizes cells and belongs to a broadly distributed protein family. mSphere 6:e0031821. doi:10.1128/mSphere.00318-21 34287011 PMC8386463

[52] O’Leary NA, Wright MW, Brister JR, Ciufo S, Haddad D, McVeigh R, Rajput B, Robbertse B, Smith-White B, Ako-Adjei D, et al.. 2016. Reference sequence (RefSeq) database at NCBI: current status, taxonomic expansion, and functional annotation. Nucleic Acids Res 44:D733–D745. doi:10.1093/nar/gkv1189 26553804 PMC4702849

[53] Sayers EW, Beck J, Brister JR, Bolton EE, Canese K, Comeau DC, Funk K, Ketter A, Kim S, Kimchi A, Kitts PA, Kuznetsov A, Lathrop S, Lu Z, McGarvey K, Madden TL, Murphy TD, O’Leary N, Phan L, Schneider VA, Thibaud-Nissen F, Trawick BW, Pruitt KD, Ostell J. 2020. Database resources of the national center for biotechnology information. Nucleic Acids Res 48:D9–D16. doi:10.1093/nar/gkz899 31602479 PMC6943063

[54] Bayer-Santos E, Lima LDP, Ceseti L de M, Ratagami CY, de Santana ES, da Silva AM, Farah CS, Alvarez-Martinez CE. 2018. Xanthomonas citri T6SS mediates resistance to Dictyostelium predation and is regulated by an ECF σ factor and cognate Ser/Thr kinase. Environ Microbiol 20:1562–1575. doi:10.1111/1462-2920.14085 29488354

[55] Spiewak HL, Shastri S, Zhang L, Schwager S, Eberl L, Vergunst AC, Thomas MS. 2019. Burkholderia cenocepacia utilizes a type VI secretion system for bacterial competition. Microbiologyopen 8:e00774. doi:10.1002/mbo3.774 30628184 PMC6612558

[56] Lewis JM, Deveson Lucas D, Harper M, Boyce JD. 2019. Systematic identification and analysis of Acinetobacter baumannii type VI secretion system effector and immunity components. Front Microbiol 10:2440. doi:10.3389/fmicb.2019.02440 31736890 PMC6833914

[57] Unterweger D, Kostiuk B, Ötjengerdes R, Wilton A, Diaz-Satizabal L, Pukatzki S. 2015. Chimeric adaptor proteins translocate diverse type VI secretion system effectors in Vibrio cholerae. EMBO J 34:2198–2210. doi:10.15252/embj.201591163 26194724 PMC4557670

[58] Crisan CV, Chande AT, Williams K, Raghuram V, Rishishwar L, Steinbach G, Watve SS, Yunker P, Jordan IK, Hammer BK. 2019. Analysis of Vibrio cholerae genomes identifies new type VI secretion system gene clusters. Genome Biol 20:163. doi:10.1186/s13059-019-1765-5 31405375 PMC6691524

[59] Bernal P, Allsopp LP, Filloux A, Llamas MA. 2017. The Pseudomonas putida T6SS is a plant warden against phytopathogens. ISME J 11:972–987. doi:10.1038/ismej.2016.169 28045455 PMC5363822

[60] Pukatzki S, Ma AT, Sturtevant D, Krastins B, Sarracino D, Nelson WC, Heidelberg JF, Mekalanos JJ. 2006. Identification of a conserved bacterial protein secretion system in Vibrio cholerae using the Dictyostelium host model system. Proc Natl Acad Sci U S A 103:1528–1533. doi:10.1073/pnas.0510322103 16432199 PMC1345711

[61] Bernardy EE, Raghuram V, Goldberg JB. 2022. Staphylococcus aureus and Pseudomonas aeruginosa isolates from the same cystic fibrosis respiratory sample coexist in coculture. Microbiol Spectr 10:e0097622. doi:10.1128/spectrum.00976-22 35867391 PMC9431432

[62] Bernardy EE, Petit RA, Raghuram V, Alexander AM, Read TD, Goldberg JB. 2020. Genotypic and phenotypic diversity of Staphylococcus aureus isolates from cystic fibrosis patient lung infections and their interactions with Pseudomonas aeruginosa. mBio 11:e00735-20. doi:10.1128/mBio.00735-20 32576671 PMC7315118

[63] Sundermann AJ, Chen J, Kumar P, Ayres AM, Cho ST, Ezeonwuka C, Griffith MP, Miller JK, Mustapha MM, Pasculle AW, Saul MI, Shutt KA, Srinivasa V, Waggle K, Snyder DJ, Cooper VS, Van Tyne D, Snyder GM, Marsh JW, Dubrawski A, Roberts MS, Harrison LH. 2022. Whole-genome sequencing surveillance and machine learning of the electronic health record for enhanced healthcare outbreak detection. Clin Infect Dis 75:476–482. doi:10.1093/cid/ciab946 34791136 PMC9427134

[64] Arndt D, Grant JR, Marcu A, Sajed T, Pon A, Liang Y, Wishart DS. 2016. PHASTER: a better, faster version of the PHAST phage search tool. Nucleic Acids Res 44:W16–W21. doi:10.1093/nar/gkw387 27141966 PMC4987931

[65] Niehus R, Oliveira NM, Li A, Fletcher AG, Foster KR. 2021. The evolution of strategy in bacterial warfare via the regulation of bacteriocins and antibiotics. Elife 10:e69756. doi:10.7554/eLife.69756 34488940 PMC8423443

[66] Mustapha MM, Srinivasa VR, Griffith MP, Cho S-T, Evans DR, Waggle K, Ezeonwuka C, Snyder DJ, Marsh JW, Harrison LH, Cooper VS, Van Tyne D. 2022. Genomic diversity of hospital-acquired infections revealed through prospective whole-genome sequencing-based surveillance. mSystems 7:e0138421. doi:10.1128/msystems.01384-21 35695507 PMC9238379

[67] Hersch SJ, Watanabe N, Stietz MS, Manera K, Kamal F, Burkinshaw B, Lam L, Pun A, Li M, Savchenko A, Dong TG. 2020. Envelope stress responses defend against type six secretion system attacks independently of immunity proteins. Nat Microbiol 5:706–714. doi:10.1038/s41564-020-0672-6 32094588 PMC7190449

[68] Le N-H, Pinedo V, Lopez J, Cava F, Feldman MF. 2021. Killing of Gram-negative and Gram-positive bacteria by a bifunctional cell wall-targeting T6SS effector. Proc Natl Acad Sci U S A 118:e2106555118. doi:10.1073/pnas.2106555118 34588306 PMC8501793

[69] Shalom G, Shaw JG, Thomas MS. 2007. In vivo expression technology identifies a type VI secretion system locus in Burkholderia pseudomallei that is induced upon invasion of macrophages. Microbiology (Reading) 153:2689–2699. doi:10.1099/mic.0.2007/006585-0 17660433

[70] Crisan CV, Hammer BK. 2020. The Vibrio cholerae type VI secretion system: toxins, regulators and consequences. Environ Microbiol 22:4112–4122. doi:10.1111/1462-2920.14976 32133757

[71] Schwarz S, Singh P, Robertson JD, LeRoux M, Skerrett SJ, Goodlett DR, West TE, Mougous JD. 2014. VgrG-5 is a Burkholderia type VI secretion system-exported protein required for multinucleated giant cell formation and virulence. Infect Immun 82:1445–1452. doi:10.1128/IAI.01368-13 24452686 PMC3993412

[72] Si M, Zhao C, Burkinshaw B, Zhang B, Wei D, Wang Y, Dong TG, Shen X. 2017. Manganese scavenging and oxidative stress response mediated by type VI secretion system in Burkholderia thailandensis. Proc Natl Acad Sci U S A 114:E2233–E2242. doi:10.1073/pnas.1614902114 28242693 PMC5358365

[73] Schwarz S, West TE, Boyer F, Chiang W-C, Carl MA, Hood RD, Rohmer L, Tolker-Nielsen T, Skerrett SJ, Mougous JD, Christie PJ. 2010. Burkholderia type VI secretion systems have distinct roles in eukaryotic and bacterial cell interactions. PLoS Pathog 6:e1001068. doi:10.1371/journal.ppat.1001068 20865170 PMC2928800

[74] Edgar RC. 2004. MUSCLE: multiple sequence alignment with high accuracy and high throughput. Nucleic Acids Res 32:1792–1797. doi:10.1093/nar/gkh340 15034147 PMC390337

[75] Guindon S, Dufayard J-F, Lefort V, Anisimova M, Hordijk W, Gascuel O. 2010. New algorithms and methods to estimate maximum-likelihood phylogenies: assessing the performance of PhyML 3.0. Syst Biol 59:307–321. doi:10.1093/sysbio/syq010 20525638

[76] Lefort V, Longueville JE, Gascuel O. 2017. SMS: smart model selection in PhyML. Mol Biol Evol 34:2422–2424. doi:10.1093/molbev/msx149 28472384 PMC5850602

[77] Letunic I, Bork P. 2021. Interactive tree of life (iTOL) v5: an online tool for phylogenetic tree display and annotation. Nucleic Acids Res 49:W293–W296. doi:10.1093/nar/gkab301 33885785 PMC8265157

[78] Jain C, Rodriguez-R LM, Phillippy AM, Konstantinidis KT, Aluru S. 2018. High throughput ANI analysis of 90K prokaryotic genomes reveals clear species boundaries. Nat Commun 9:5114. doi:10.1038/s41467-018-07641-9 30504855 PMC6269478

[79] Welker E, Domfeh Y, Tyagi D, Sinha S, Fisher N. 2015. Genetic manipulation of Stenotrophomonas maltophilia. Curr Protoc Microbiol 37:6F. doi:10.1002/9780471729259.mc06f02s37 PMC4562062 26344220

[80] Davies DG, Parsek MR, Pearson JP, Iglewski BH, Costerton JW, Greenberg EP. 1998. The involvement of cell-to-cell signals in the development of a bacterial biofilm. Science 280:295–298. doi:10.1126/science.280.5361.295 9535661

[81] Lagendijk EL, Validov S, Lamers GEM, de Weert S, Bloemberg GV. 2010. Genetic tools for tagging Gram-negative bacteria with mCherry for visualization in vitro and in natural habitats, biofilm and pathogenicity studies. FEMS Microbiol Lett 305:81–90. doi:10.1111/j.1574-6968.2010.01916.x 20180857

