# Supplementary material for: The type VI secretion system of the emerging pathogen Stenotrophomonas maltophilia complex has antibacterial properties
Source: mSphere. 2023 Nov 17;8(6):e00584-23. doi: 10.1128/msphere.00584-23 (PMC10732056; doi:10.1128/msphere.00584-23)
Supplement: Supplemental Figures — Figures S1-S4. [file msphere.00584-23-s0001.docx]

**SUPPLEMENTARY FIGURES**

**
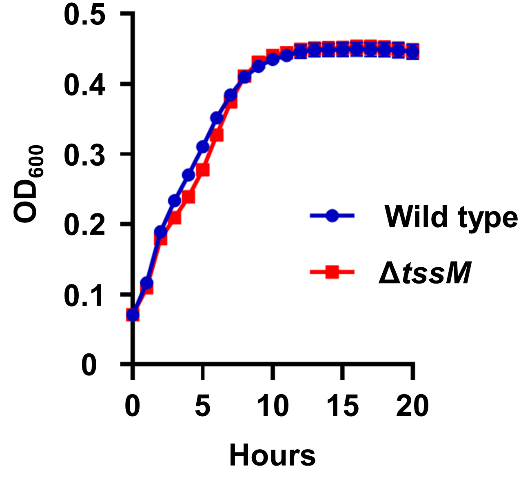
**

**Supplementary Figure 1. WT and Δ*tssM* *S. maltophilia* strains have similar growth in liquid LB medium.** WT and T6SS *S. maltophilia* overnight cultures were back-diluted to an OD_600_ ≈ 0.1 in fresh LB and incubated at 37° with shaking in 96-well plates. OD_600_ readings were taken using a BioTek Synergy H1 spectrophotometer. Results from four independent experiments are shown.

**
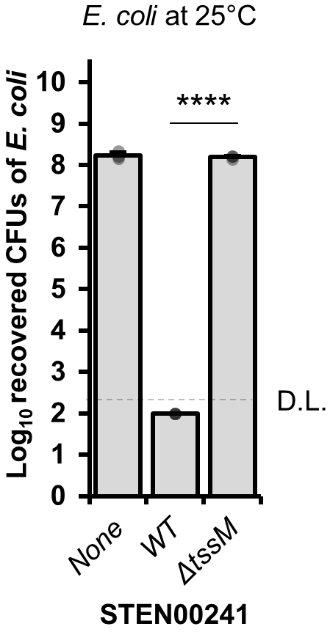
**

**Supplementary Figure 2. The *S. maltophilia* T6SS is required for killing *E. coli* at 25°C.** *E. coli* resistant to tetracycline was grown alone or in the presence of *S. maltophilia* (WT or Δ*tssM*) on solid LB medium for 20 hours at 25°C. The number of surviving *E. coli* cells was determined by plating mixtures on tetracycline plates. Three independent biological replicates were performed. A one-way ANOVA with post-hoc Tukey HSD was used to determine statistical significance. NS – not significant (p > 0.05). D.L. – detection limit.

**
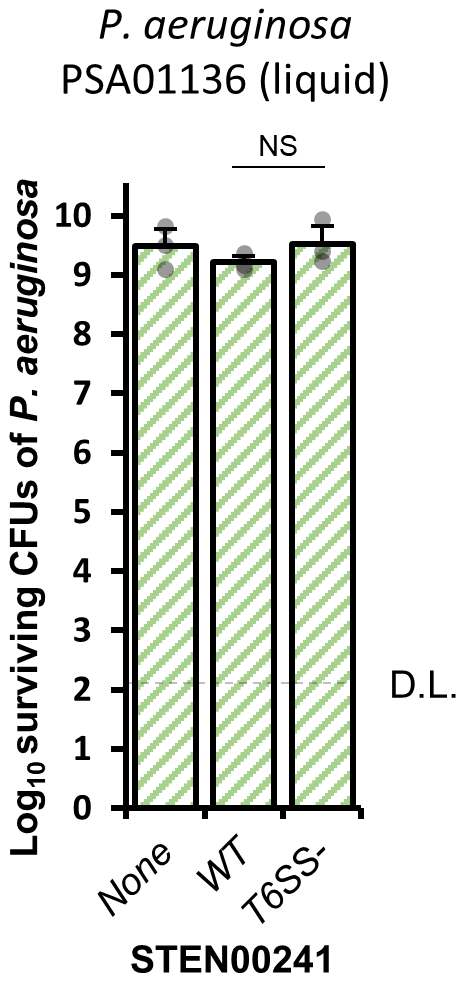
**

**Supplementary Figure 3. The *S. maltophilia* T6SS is not effective at eliminating *P. aeruginosa* PSA01136 in liquid LB medium.** *P. aeruginosa* PSA01136 was grown alone or in the presence of *S. maltophilia* (WT or Δ*tssM*) in liquid LB medium and incubated for 20 hours at 37°C. The number of surviving *P. aeruginosa* PSA01136 cells was determined by plating mixtures on chloramphenicol plates. Three independent biological replicates were performed. A one-way ANOVA with post-hoc Tukey HSD was used to determine statistical significance. NS – not significant (p > 0.05). D.L. – detection limit.

**
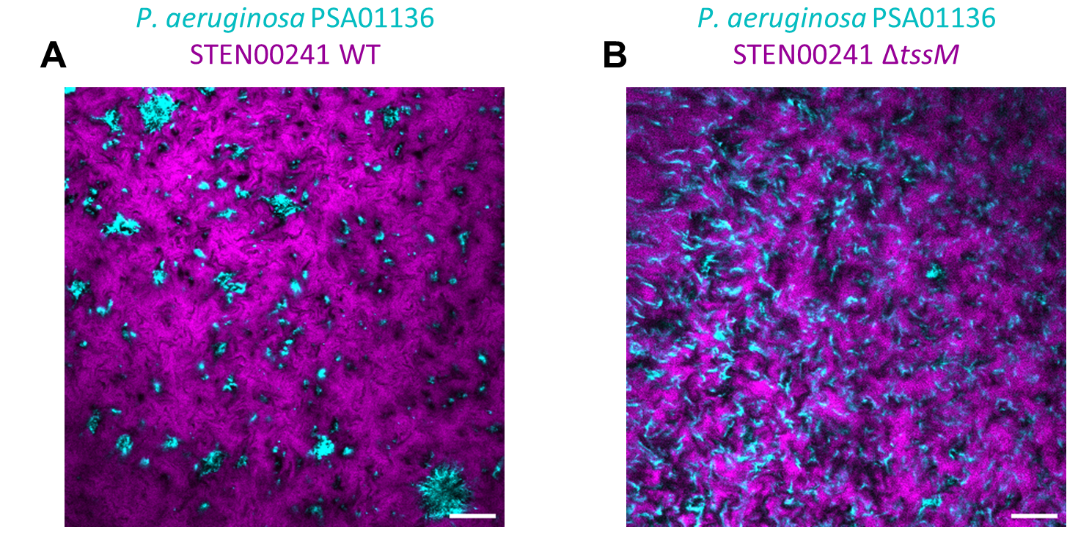
**

**Supplementary Figure 4. Different biological replicate of the co-cultures between STEN00241 (colored here with magenta) and PSA01136 (colored here with cyan).** Co-cultures between *P. aeruginosa* PSA01136 expressing GFP (colored with cyan) and STEN00241 WT expressing mCherry (colored with magenta) (A), or co-cultures between *P. aeruginosa* PSA01136 expressing GFP (colored with cyan) and *S. maltophilia* Δ*tssM* expressing mCherry (colored with magenta) (F) spotted onto LB plates were visualized using a Zeiss LSM 710 upright microscope without a cover slip. Scale bar represents 100 µm.
