# Supplementary material for: The type VI secretion system of the emerging pathogen Stenotrophomonas maltophilia complex has antibacterial properties
Source: mSphere. 2023 Nov 17;8(6):e00584-23. doi: 10.1128/msphere.00584-23 (PMC10732056; doi:10.1128/msphere.00584-23)
Supplement: Supplemental Tables — Tables S1-S3. [file msphere.00584-23-s0002.docx]

**Supplementary Table 1. TssC accession numbers and *Stenotrophomonas maltophilia* complex strain details.**

| **Protein Accession Number** | **Stain Name** | **Assembly** | **Country** | **Source** | **Year** |
| --- | --- | --- | --- | --- | --- |
| VUP75632.1 | H_144_saintdenis | GCA_902158055.1 | France | Human urine | 2019 |
| WP_110714162.1 | AS012746 | GCF_010604305.1 | USA | Human lung | 2015 |
| VUR71899.1 | H_59_creteil | GCA_902158415.1 | France | Sputum from cystic fibrosis patient | 2019 |
| VUN66964.1 | A1_185015_oise | GCA_902157465.1 | France | Horse tracheal wash | 2019 |
| VUN37614.1 | A1_184926_mayenne | GCA_902157335.1 | France | Horse tracheal wash | 2019 |
| VUN17743.1 | A1_183848_loiret | GCA_902157295.1 | France | Horse tracheal wash | 2019 |
| VUM68104.1 | A1_179847_maineetloire | GCA_902157155.1 | France | Horse tracheal wash | 2019 |
| VUM64612.1 | A1_179881_eure | GCA_902157175.1 | France | Horse tracheal wash | 2019 |
| VUI45012.1 | A1_123013_yvelines | GCA_902156375.1 | France | Horse tracheal wash | 2019 |
| UQA70471.1 | FZD2 | GCA_023277525.1 | Poland | Activated Sludge | 2018 |
| RTQ90998.1 | MDMC339 | GCA_003970865.1 | Morocco | Desert Sand | 2002 |
| MBH1619136.1 | STEN00145 | GCA_015997495.1 | USA | Human sputum | 2018 |
| MBA0429346.1 | 4834-R | GCA_013464455.1 | Spain | Human | 1991 |
| MBA0361510.1 | EV5520 | GCA_013463805.1 | Spain | Environmental control | 2011 |
| MBA0324222.1 | PU109 | GCA_013463885.1 | Belgium | Human perineum swab | 2012 |
| MBA0286468.1 | OU110 | GCA_013463855.1 | Belgium | Human Oropharynx | 2012 |
| AWT16776.1 | SJTL3 | GCA_003205835.1 | China | Wastewater | 2017 |
| MBH1430467.1 | STEN00182 | GCA_015996945.1 | USA | Human sputum | 2018 |
| MBH1599719.1 | STEN00137 | GCA_015997675.1 | USA | Human blood | 2018 |
| MBH1556142.1 | STEN00146 | GCA_015997485.1 | USA | Human sputum | 2018 |
| PZP87459.1 | S2_009_000_R1_70 | GCA_003241275.1 | USA | Hospital neonatal intensive care unit surfaces and sink samples | 2014 |
| WP_006465400.1 | AS012638 | GCF_010608115.1 | USA | Human lung | 2015 |
| VUO77094.1 | H_102_saintnazaire | GCA_902157675.1 | France | Human sputum | 2019 |
| MBS4803210.1 | L3_114_000G1_dasL3_114_000G1_metabat.metabat.34 | GCA_018363575.1 | USA | Human feces | 2016/ 2019 |
| MBA0437178.1 | L9-5R5 | GCA_013464495.1 | Spain | Human | 1999 |
| CCP12928.1 | SKK35 | GCA_000355745.1 | Germany | Human ulcer swab | 2013 |
| WP_040007501.1 | AS012684 | GCF_010605095.1 | USA | Human lung | 2016 |
| VUQ196240.1 | H_160_stpierre | GCA_902158105.1 | France | Human sputum | 2019 |
| VUP22537.1 | H_107_bayonne | GCA_902157615.1 | France | Human tracheal/bronchial aspiration | 2019 |
| VUO05089.1 | A1_186526_vienne | GCA_902157415.1 | France | Horse tracheal wash | 2019 |
| VUN09045.1 | A1_175746_seinemaritime | GCA_902157215.1 | France | Horse transtracheal aspiration | 2019 |
| VUN07624.1 | A1_175792_seinemaritime | GCA_902157195.1 | France | Horse transtracheal aspiration | 2019 |
| VUM76323.1 | A1_179962_valdoise | GCA_902157255.1 | France | Horse tracheal wash | 2019 |
| VUJ75540.1 | A1_133498_basrhin | GCA_902156525.1 | France | Horse transtracheal aspiration | 2019 |
| VUJ50913.1 | A1_134082_basrhin | GCA_902156535.1 | France | Horse transtracheal aspiration | 2019 |
| MBH1788207.1 | STEN00049 | GCA_015998575.1 | USA | Human sputum | 2017 |
| MBH1602795.1 | STEN00137 | GCA_015997675.1 | USA | Human blood | 2018 |
| MBH1556878.1 | STEN00146 | GCA_015997485.1 | USA | Human sputum | 2018 |
| MBH1505173.1 | STEN00166 | GCA_015997245.1 | USA | Human wound | 2018 |
| MBH1479980.1 | STEN00173 | GCA_015997065.1 | USA | Human wound | 2018 |
| MBH1365939.1 | STEN00203 | GCA_015996535.1 | USA | Human sputum | 2018 |
| KOQ71993.1 | B5 | GCA_001276385.1 | Australia | Lungs of cystic fibrosis patient | 2005 |
| ALA82908.1 | ISMMS3 | GCA_001274595.1 | USA | Human blood | 2013 |
| WP_079222223.1 | AA1 | GCF_002025605.1 | USA | Zea mays root | 2012 |
| MBH1509406.1 | STEN00164 | GCA_015997185.1 | USA | Human sputum | 2018 |
| WP_110712666.1 | AS012746 | GCF_010604305.1 | USA | Human lung | 2015 |
| VUR90417.1 | H_59_creteil | GCA_902158415.1 | France | Sputum from cystic fibrosis patient | 2019 |
| VUQ21281.1 | H_144_saintdenis | GCA_902158055.1 | France | Human urine | 2019 |
| VUN91742.1 | A1_185015_oise | GCA_902157465.1 | France | Horse tracheal wash | 2019 |
| VUN57443.1 | A1_184926_mayenne | GCA_902157335.1 | France | Horse tracheal wash | 2019 |
| VUN42780.1 | A1_183848_loiret | GCA_902157295.1 | France | Horse tracheal wash | 2019 |
| VUN19319.1 | A1_179847_maineetloire | GCA_902157155.1 | France | Horse tracheal wash | 2019 |
| VUM97619.1 | A1_179881_eure | GCA_902157175.1 | France | Horse tracheal wash | 2019 |
| UQA68845.1 | FZD2 | GCA_023277525.1 | Poland | Activated Sludge | 2018 |
| MBH1619198.1 | STEN00145 | GCA_015997495.1 | USA | Human sputum | 2018 |
| MBA0448754.1 | E77 | GCA_013464485.1 | Spain | Human sputum | 2009 |
| MBA0430392.1 | 4834-R | GCA_013464455.1 | Spain | Human | 1999 |
| MBA0361851.1 | EV5520 | GCA_013463805.1 | Spain | Environmental control | 2011 |
| MBA0322458.1 | PU109 | GCA_013463885.1 | Belgium | Human perineum swab | 2012 |
| MBA0283922.1 | OU110 | GCA_013463855.1 | Belgium | Human Oropharynx | 2012 |
| AWT15240.1 | SJTL3 | GCA_003205835.1 | China | Wastewater | 2017 |
| KRG50788.1 | LMG 978 | GCA_001431665.1 | Sri Lanka | Piper betle | 1928 |
| WP_164072634.1 | AS012546 | GCF_010587625.1 | USA | Human lung | 2015 |
| UQY96326.1 | GYH | GCA_023518235.1 | China | Unknown | 2019 |
| MBN5027172.1 | STEN00241 | GCA_017150115.1 | USA | Human sputum | 2019 |
| WP_053517619.1 | AS012568 | GCF_010609345.1 | USA | Human lung | 2015 |
| VUQ30377.1 | H_146_roubaix | GCA_902157995.1 | France | Human Blood | 2019 |
| VUP95626.1 | H_152_versailles | GCA_902158025.1 | France | Human protected specimen brush | 2019 |
| MBH1665108.1 | STEN00084 | GCA_015997955.1 | USA | Human urine | 2018 |
| MBA0223583.1 | OU111 | GCA_013463485.1 | Belgium | Human oropharynx | 2012 |
| KOQ69818.1 | B4 | GCA_001276355.1 | Australia | Lungs of cystic fibrosis patient | 2005 |
| MBH1592951.1 | STEN00085 | GCA_015998015.1 | USA | Human bronch | 2018 |
| MBA0484470.1 | PC272 | GCA_013464675.1 | Germany | Human perineum swab | 2012 |
| MBA0477851.1 | PC273 | GCA_013464645.1 | Germany | Human perineum swab | 2012 |
| MBA0467865.1 | PC271 | GCA_013464685.1 | Germany | Human perineum swab | 2012 |
| MBA0264340.1 | PC274 | GCA_013463605.1 | Germany | Human perineum swab | 2012 |
| MBH1866844.1 | STEN00030 | GCA_015998895.1 | USA | Human sputum | 2017 |
| WP_182267168.1 | T50-20 | GCF_014076535.1 | China | Biofilm reactor | 2018 |

**Supplementary Table 2. Strains and plasmids used in this study.**

| **Strains** | **Genotype** | **Source** |
| --- | --- | --- |
| *Escherichia coli* DH5α | Wild type | Invitrogen |
| *Escherichia coli* SM10 | Wild type | (1) |
| *Pseudomonas aeruginosa* PA14 | Wild type | (2) |
| *Pseudomonas aeruginosa* PA32 | Wild type | (3) |
| *Pseudomonas aeruginosa* PSA01136 | Wild type | (4) |
| *Stenotrophomonas maltophilia* complex STEN00241 | Wild type | (4) |
| CCV081 | *Stenotrophomonas maltophilia* complex STEN00241 Δ*tssM* | This study |
| *Staphylococcus aureus* JE2 | Wild type | (5) |
| *Burkholderia cenocepacia* K56-2 | Wild type | (6) |
| **Plasmid** | **Description** | **Source** |
| pEX18Tc | Suicide vector to engineer genetic mutations | (7) |
| pMRP9-1 (GFP+) | Constitutive expression of GFP | (8) |
| pMP7605 (mCherry+) | Constitutive expression of mCherry | (9) |
| pCCV077 (pEX18Tc-Δ*tssM*) | Used to create the *Stenotrophomonas maltophilia* complex STEN00241 Δ*tssM* strain | This study |

1. Simon R, Priefer U, Pühler A. 1983. A Broad Host Range Mobilization System for In Vivo Genetic Engineering: Transposon Mutagenesis in Gram Negative Bacteria. Bio/Technology 1:784–791.

2. Rahme LG, Stevens EJ, Wolfort SF, Shao J, Tompkins RG, Ausubel FM. 1995. Common Virulence Factors for Bacterial Pathogenicity in Plants and Animals. Science 268:1899–1902.

3. Cross AR, Goldberg JB. 2019. Remodeling of O Antigen in Mucoid *Pseudomonas aeruginosa* via Transcriptional Repression of wzz2. MBio 10.

4. Sundermann AJ, Chen J, Kumar P, Ayres AM, Cho S-T, Ezeonwuka C, Griffith MP, Miller JK, Mustapha MM, Pasculle AW, Saul MI, Shutt KA, Srinivasa V, Waggle K, Snyder DJ, Cooper VS, Van Tyne D, Snyder GM, Marsh JW, Dubrawski A, Roberts MS, Harrison LH. 2021. Whole-Genome Sequencing Surveillance and Machine Learning of the Electronic Health Record for Enhanced Healthcare Outbreak Detection. Clin Infect Dis https://doi.org/10.1093/cid/ciab946.

5. Kennedy AD, Otto M, Braughton KR, Whitney AR, Chen L, Mathema B, Mediavilla JR, Byrne KA, Parkins LD, Tenover FC, Kreiswirth BN, Musser JM, DeLeo FR. 2008. Epidemic community-associated methicillin-resistant *Staphylococcus aureus* : Recent clonal expansion and diversification. Proc Natl Acad Sci 105:1327–1332.

6. Darling P, Chan M, Cox AD, Sokol PA. 1998. Siderophore Production by Cystic Fibrosis Isolates of *Burkholderia cepacia*. Infect Immun 66:874–877.

7. Hoang TT, Karkhoff-Schweizer RR, Kutchma AJ, Schweizer HP. 1998. A broad-host-range Flp-FRT recombination system for site-specific excision of chromosomally-located DNA sequences: application for isolation of unmarked *Pseudomonas aeruginosa* mutants. Gene 212:77–86.

8. Davies DG, Parsek MR, Pearson JP, Iglewski BH, Costerton JW, Greenberg EP. 1998. The Involvement of Cell-to-Cell Signals in the Development of a Bacterial Biofilm. Science 280:295–298.

9. Lagendijk EL, Validov S, Lamers GEM, De Weert S, Bloemberg G V. 2010. Genetic tools for tagging Gram-negative bacteria with mCherry for visualization in vitro and in natural habitats, biofilm and pathogenicity studies. FEMS Microbiol Lett 305:81–90.

**Supplementary Table 3. Primers used in this study.**

| **Name** | **Sequence (5’ - 3’)** | **Description** |
| --- | --- | --- |
| oCCV007 | taaaacgacggccagtgccagcaggtgcgccagga | Forward primer to amplify 1000bp upstream of *tssM* from STEN00241 for the pEX18Tc *tssM* deletion construct |
| oCCV008 | ggcactgaccacccggctcacatgcagggatccaggg | Reverse primer to amplify 1000bp upstream of *tssM* from STEN00241 for the pEX18Tc *tssM* deletion construct |
| oCCV009 | gacccctggatccctgcatgtgagccgggtggtcag | Forward primer to amplify 1000bp downstream of *tssM* from STEN00241 for the pEX18Tc *tssM* deletion construct |
| oCCV010 | gtacccggggatcctctagagattgcacaggcgcatcg | Reverse primer to amplify 1000bp downstream of *tssM* from STEN00241 for the pEX18Tc *tssM* deletion construct |
